# Effects of Continuous Postoperative Pericardial FLUshing with Investigational Device on Postoperative Re-Explorations for Bleeding (FLUID)—Randomized Clinical Trial

**DOI:** 10.3390/jcm15062151

**Published:** 2026-03-11

**Authors:** Manon A. Molenaar, Dave R. Koolbergen, Martijn Vegter, Kayan Lam, Frederik N. Hofman, Stefan R. van Dinter, Annette van ‘t Loo, Arend de Weger, Jeroen A. Janson, Carolien S. E. Bulte, Susanne Eberl, Wim K. Lagrand, Alexander Vonk, Felice R. M. Lucas, Robert J. M. Klautz, Marcus J. Schultz

**Affiliations:** 1 Department of Intensive Care, Amsterdam University Medical Centers, 1105 AZ Amsterdam, The Netherlands; 2 Department of Cardiothoracic Surgery, Amsterdam University Medical Centers, 1105 AZ Amsterdam, The Netherlands; 3 Department of Cardiothoracic Surgery, Leiden University Medical Center, 2333 ZG Leiden, The Netherlands; 4 Department of Cardiothoracic Surgery, Catharina Hospital, 5623 EJ Eindhoven, The Netherlands; 5 Department of Cardiothoracic Surgery, St. Antonius Hospital, 3435 CM Nieuwegein, The Netherlands; 6 Department of Intensive Care, Leiden University Medical Center, 2333 ZG Leiden, The Netherlands; 7 Department of Anesthesiology, Amsterdam University Medical Centers, 1105 AZ Amsterdam, The Netherlands

**Keywords:** cardiac surgery, bleeding, tamponade, pericardial flushing, surgical re-exploration

## Abstract

**Objectives:** Continuous postoperative pericardial flushing (CPPF) may prevent postoperative re-explorations for tamponade or excessive postoperative bleeding due to a non-surgical cause in cardiac surgery patients. **Methods:** An investigator-initiated, national, multicenter, randomized clinical superiority trial was performed in four hospitals in the Netherlands between November 2021 and April 2023. Patients undergoing general cardiac surgery involving cardiopulmonary bypass were randomly assigned to receive CPPF or standard care. The primary endpoint was the number of surgical re-explorations for either cardiac tamponade or excessive postoperative bleeding due to a non-surgical cause in the first postoperative week. **Results:** The study was stopped after randomization of 164 patients, of which 79 were allocated to CPPF and 85 served as controls. The number of surgical re-explorations for either cardiac tamponade or excessive postoperative bleeding due to a non-surgical cause in the first postoperative week was not different between CPPF patients and control patients (3.8% vs. 2.4%; relative risk 1.61 [0.28–9.41]; *p* = 0.67). There were no safety issues related to use of the investigational device. **Conclusions:** In this prematurely stopped study, CPPF did not reduce the number of postoperative re-explorations for cardiac tamponade or excessive bleeding due to a non-surgical cause in cardiac surgery patients. Additional well-powered studies remain needed to determine whether CPPF has any beneficial effect on outcome.

## 1. Introduction

A significant postoperative complication following cardiac surgery is excessive and prolonged bleeding, which may require surgical re-exploration because of cardiac tamponade or hemodynamic instability. A surgical re-exploration substantially affects patient recovery and overall outcomes, leading to increased mortality rates, along with prolonged hospitalization [1,2,3,4,5,6].

Excessive or prolonged postoperative bleeding may be prevented by continuous postoperative pericardial flushing (CPPF). CPPF involves the gentle irrigation of the pericardial space with a warm saline solution during the initial postoperative hours. This intervention aims to dilute blood and clots, facilitating their evacuation from the pericardial space and preventing chest tube obstruction [7,8,9,10,11,12,13,14]. Current standard practice involves the placement of mediastinal chest tubes to drain excess blood and fluids. However, these tubes frequently become obstructed, resulting in the accumulation of blood and clots in the pericardial space.

In contrast to other techniques to improve pericardial drainage, CPPF is an integrated approach. It enhances chest tube patency through continuous flushing and dilutes blood and clots in the pericardial space to promote drainage. In addition, the rationale for CPPF is supported by a commonly observed clinical phenomenon during surgical re-explorations, in which pericardial irrigation with a warm saline solution and removal of clots is sufficient to stop the bleeding [15]. Notably, three prior single-center clinical studies have confirmed the safety and feasibility of CPPF and demonstrated a significant reduction in postoperative blood loss in cardiac surgery patients [16,17,18]. However, these studies were limited by their sample size and single-center design; the flushing was performed by a researcher rather than by a dedicated medical device; and the studies were not powered to detect clinically relevant endpoints, such as postoperative re-explorations.

We conducted a randomized clinical trial, named ‘FLUsh with Investigational Device’ (FLUID), with the aim to determine the effects of CPPF, performed with the Haermonics investigational device, on the incidence of re-explorations in patients that underwent cardiac surgery with the use of cardiopulmonary bypass. Our hypothesis was that CPPF is superior to standard care alone in preventing surgical re-exploration for either cardiac tamponade or excessive postoperative bleeding due to a non-surgical cause.

## 2. Materials and Methods

### 2.1. Study Design

FLUID was an investigator-initiated, national, multicenter, randomized clinical superiority trial performed at four hospitals: Amsterdam University Medical Centers, location ‘AMC’, Amsterdam; Leiden University Medical Center, Leiden; Catharina Hospital, Eindhoven; and St. Antonius Hospital, Nieuwegein, The Netherlands. The study protocol of FLUID was first approved by the Institutional Review Board of the Amsterdam University Medical Center, location AMC (2021_057), and thereafter in the other three hospitals. Written informed consent was obtained from all patients before surgery. The study was registered at clinicaltrials.gov (identifier NCT05308589).

### 2.2. Patients

Patients were eligible for participation if: (1) scheduled for coronary artery bypass grafting with continued dual antiplatelet therapy (DAPT); (2) scheduled for valve surgery due to acute infective endocarditis; (3) scheduled for complex or multiple cardiac (redo) procedures with an (expected) cardiopulmonary bypass time of >300 min; or (4) undergoing aortic surgery with deep hypothermic circulatory arrest (DHCA) and (5) having provided written informed consent. During the conduct of the study, changes in perioperative management and treatment protocols for DAPT resulted in a reduction in the initial study population, namely patients undergoing coronary artery bypass grafting (CABG) with continued DAPT. Consequently, the inclusion criteria were broadened from December 2022 onwards to allow the inclusion of patients that were operated on without continued DAPT.

The exclusion criteria were: age < 18 years; Euroscore II > 20%; emergency surgery; off-pump surgery or minimally invasive cardiac surgery (e.g., mini-thoracotomy); participation in another interventional study; and intraoperative diaphragm injury.

### 2.3. Investigational Device

The Haermonics investigational device was built with off-the-shelf components (Supplementary Figure S1) configured to achieve four essential functionalities: (1) automatic monitoring of the outflow volume; (2) quantification of the content of the outflow volume by means of real-time and continuous hematocrit analysis of the MCTD; (3) warming of the flushing fluid to body temperature; and (4) continuous intrapericardial pressure measurement. The device flushes the peri-surgical spaces, i.e., the pericardial, pleural and mediastinal spaces, after cardio-thoracic surgery with the intention to reduce postoperative bleeding and postoperative complications related to bleeding or coagulation. The device works by continuous flushing of the peri-surgical space via drainage with a saline rinsing solution to enhance the evacuation of blood and clots in the pericardial cavity and mediastinum. Use of the device may also reduce clogging of the chest tubes, thereby maintaining patency of the drainage and preventing subsequent accumulation of blood and clots in the pericardial space.

Preparation and labelling of the device prior to use was performed on the day of surgery. Outside the operating room, the device was tested for full functionality by verifying that the battery was fully charged and that the infusion warming device and volumetric pump were properly working. The required disposables were unpacked and installed; if the sterility was broken before use, all the disposables were replaced. The device was connected to the mobile vacuum unit, after which the system was primed, then all sensors were tested for full functionality. Inside the operating room, the sterile parts of the inflow tube and outflow drain were passed to the scrub nurse to be connected to the inflow tube positioned in the pericardial space and the Y-piece on the chest tubes, respectively.

### 2.4. Standard Care

Care was provided following local guidelines for cardiac surgery. Control patients, randomized to receive standard care alone, were equipped with one draining tube in the pericardial space and another in the anterior mediastinum at the conclusion of surgery. Additionally, each surgically opened pleural cavity was drained separately with an additional tube. The draining tubes were connected to each other with the use of a Y-piece. After surgery, when postoperative bleeding had stopped, all chest tubes were removed.

### 2.5. Intervention

Patients randomized to receive CPPF therapy with the Haermonics investigational device received one additional tube in the pericardial space through an extra incision hole at the end of surgery. This tube was connected to a bag filled with the irrigation solution (0.9% NaCl solution). The irrigation solution, heated to body temperature, was flushed through the additional tube at a fixed flow rate of 500 mL/h using a volumetric pump. CPPF started immediately and was performed continuously until a total of 4000 mL irrigation solution had been flushed through the pericardial space, i.e., CPPF lasted eight hours.

### 2.6. Randomization and Blinding

Randomization was performed at the latest possible timepoint, i.e., close to sternal closure. Patients were randomized in a 1:1 ratio, using random block sizes of four to eight patients and stratified per center. The local study team performed randomization with a password-protected, web-based randomization system (SSL-encrypted website, Castor Electronic Data Capture, Amsterdam, the Netherlands). Because of the nature of the intervention, blinding was not possible. However, the independent clinical event committee that determined the reason for each re-exploration that occurred within the first postoperative week was kept blind at all times.

### 2.7. Data Collected

The following data were collected:

(1) Patient demographics and baseline characteristics, including year of birth, sex, age, body height and weight, Euroscore II, cardiovascular risk factors, and comorbidities; the type, dose and stopping date of anticoagulant therapy before surgery; cardiac echocardiography for preoperative left ventricular function; and standard laboratory tests. (2) Surgery characteristics, including the type of surgery, including coronary artery bypass grafting (CABG), type of valve surgery, and aortic surgery; the duration of surgery, duration of cardiopulmonary bypass, and duration of cross-clamping; the number and location of drain placements and the number of surgically opened cavities; infusion of blood products; and coagulation status at the end of surgery. (3) Postoperative outcomes, including surgical re-explorations with their reasons, the hourly inflow volume and outflow drain volume in the first eight postoperative hours, and infusion of blood products until discharge from the ICU. (4) Long-time follow-up, including life status and hospital discharge at three months.

### 2.8. Definitions

An independent clinical event committee determined the reason for each re-exploration that occurred within the first postoperative week. The members had access to the surgical report and the clinical data between surgery and re-exploration. Reasons for re-explorations were scored as excessive bleeding, hemodynamic instability, and/or cardiac tamponade. In the case of bleeding, the re-explorations were scored as a result of a surgical or a non-surgical cause. Surgical bleeding was defined as postoperative bleeding with a clear surgical cause, such as bleeding at the suture site. Non-surgical bleeding was defined as postoperative bleeding without a direct surgical cause.

In CPPF patients, the hourly blood loss during the initial eight postoperative hours was determined by deducting the total infused irrigation volume from the total mediastinal chest tube drainage volume at eight hours after surgery. In cases where irrigation fluid was retained in body cavities, resulting in a ‘negative’ volume, we addressed this by adjusting the blood loss for that specific hour to zero. Blood loss data were imputed based on the last observation carried forward (LOCF) principle. In case of completely missing records of fluid balances, we did not impute and excluded the patient records from the blood loss analysis.

### 2.9. Study Endpoints

The primary endpoint was a composite endpoint: the incidence of surgical re-exploration for either cardiac tamponade or excessive postoperative bleeding due to a non-surgical cause within the first postoperative week. Surgical re-exploration for another reason did not count herein. The secondary endpoints included the two components of the primary endpoint—the number of re-explorations for cardiac tamponade or excessive bleeding due to surgical causes within the first postoperative week, as well as the number of re-explorations for other reasons than bleeding—along with blood loss within the first eight postoperative hours, transfusion of blood products until ICU discharge, new-onset postoperative atrial fibrillation (POAF) until discharge, and length of stay in the hospital for surgery. We also report minimally invasive interventions for pericardial or intrapleural fluid accumulation and mortality until day 90.

In FLUID we also validated the hematocrit and pressure sensors of the investigational device. These technical outcomes will be reported elsewhere.

### 2.10. Safety Assessment

Severe adverse advents were recorded in accordance with the Medical Device Regulation (MDR) using a predefined list and were independently monitored for completeness. Events were assessed for potential device-related causality. Device deficiencies were evaluated separately by the manufacturer and were not part of the present study.

### 2.11. Sample Size Calculation

FLUID was initially designed to enroll 414 patients in total (207 in each arm) using the original inclusion criteria. The sample size calculation was adjusted after broadening of the inclusion criteria to enroll 992 patients in total (496 in each arm). The sample size was calculated to detect a reduction in the proportion of re-explorations of 70% with a type I error of 5%, power of 80% and correction for 10% of dropouts, based on the findings of previous studies [16,17,18].

The study was stopped at 164 patients because of several improvements to the medical device and its usage. It was deemed necessary to start a new study that will use the improved version of the device. Thus, the study stopped prematurely, leaving us with a sample size of patients which were followed up as planned.

### 2.12. Statistical Analysis

Baseline characteristics, patient demographics and perioperative data are presented as numbers and percentages for categorical variables and medians and interquartile ranges for continuous variables.

Following an intention-to-treat analysis, the primary endpoint is presented as the number of re-explorations for either cardiac tamponade or excessive bleeding due to a non-surgical cause. Differences in the incidence of the primary outcome between both allocation arms were assessed using a 2 × 2 contingency table resulting in a risk ratio and accompanying 95% confidence interval (CI).

Secondary outcome data are presented as numbers and percentages for categorical variables and as medians and interquartile ranges for continuous variables. Categorical secondary outcomes were assessed using a 2 × 2 contingency table resulting in a risk ratio and accompanying 95% confidence interval (CI). Differences in continuous outcomes between both allocation arms were analyzed using the unpaired t-test or Mann–Whitney U test where appropriate.

We performed a per-protocol analysis wherein patients who were randomized to the CPPF group but did not receive CPPF treatment were excluded.

We performed one post hoc analysis wherein we determined the effects of CPPF on the incidence of re-explorations in patients with a high risk of bleeding. This included patients scheduled for CABG with continued dual antiplatelet therapy, patients with acute infective endocarditis scheduled for valve surgery, patients scheduled for complex or multiple cardiac procedures with a cardiopulmonary bypass time over five hours and patients undergoing aortic surgery with deep hypothermic circulatory arrest.

All analyses were performed using R software, version 4.3.2 (R Core team, Vienna, Austria). A *p*-value of <0.05 was considered statistically significant.

## 3. Results

### 3.1. Patients

**Figure 1:**
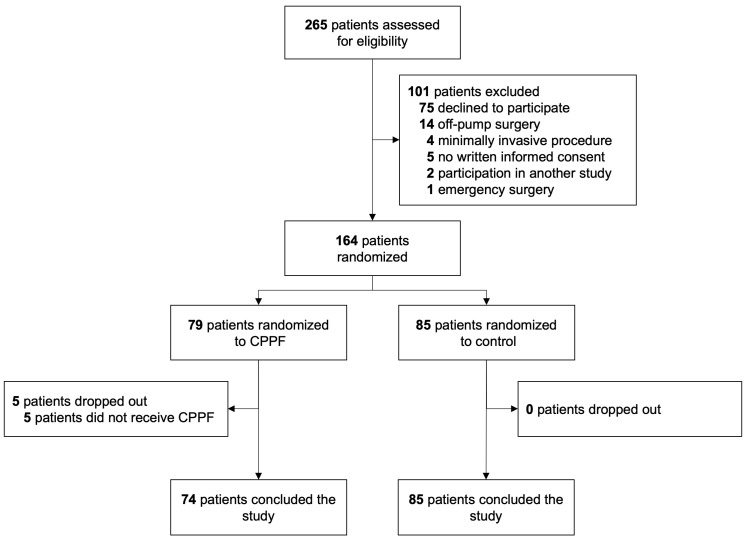
CONSORT flowchart.

Of 265 patients screened from November 2021 through April 2023, 164 patients were randomized: 79 were allocated to CPPF and 85 to standard care alone (Figure 1). The patients were predominantly male, mainly undergoing CABG or valve surgery (Table 1 and Table 2). Patient demographics and baseline characteristics, preoperative anticoagulation, and surgery characteristics were not different between the two groups (Table 2).

### 3.2. Primary Endpoint

Surgical re-explorations for either cardiac tamponade or excessive bleeding due to a non-surgical cause within the first postoperative week occurred in three patients (3.8%) in the CPPF group compared with two patients (2.4%) in the standard care group (RR 1.61 [0.28–9.41]; *p* = 0.67) (Table 3). There was no difference between the two groups in the occurrence of the two components of the primary endpoint.

### 3.3. Secondary Endpoints

Surgical re-explorations for excessive bleeding due to a surgical cause were also not different (Table 3). Compared to that in control patients, the median blood loss within the first 8 h after surgery was lower in CPPF patients (270 [40–490] vs. 355 [250–555] mL; *p* = 0.02). Re-explorations that were performed for non-bleeding-related reasons, transfusion of blood products and administration of coagulation factors until ICU discharge, new-onset postoperative atrial fibrillation until hospital discharge, hospital stay length and mortality were not different between the two groups (Table 4).

### 3.4. Per-Protocol and Post Hoc Analyses

The per-protocol (Supplementary Table S1) and post hoc analyses did not change the findings (Supplementary Table S2).

## 4. Discussion

The findings of this national, multicenter, randomized clinical superiority trial of CPPF in patients undergoing general cardiac surgery involving cardiopulmonary bypass can be summarized as follows: (1) CPPF neither prevented postoperative re-explorations for excessive non-surgical bleeding nor (2) prevented postoperative re-explorations for cardiac tamponade in cardiac surgery patients. Furthermore, (3) CPPF was associated with less postoperative blood loss; (4) CPPF did not affect the transfusion of blood products; and (5) CPPF did not affect the occurrence of postoperative atrial fibrillation.

Our study has strengths. Different from previous studies of CPPF [16,17,18], this was a multicenter randomized clinical trial involving two academic hospitals and two nonacademic teaching hospitals, contributing to its generalizability to investigate the role of CPPF in patients undergoing cardiac surgery with the use of cardiopulmonary bypass. We measured a clinically relevant patient-centered outcome. This composite endpoint was chosen because it reflects all causes of re-exploration. The study was designed to minimize bias by using concealed allocation, randomization was performed right before sternal closure, and an intention-to-treat analysis with a pragmatic protocol that was strictly adhered to. An independent blind clinical event committee determined whether the reason for each re-exploration was non-surgical or surgical bleeding, and the study had no loss to follow-up with regard to the primary endpoint. In addition, patients were enrolled in the trial over a period of two years, during which standardized care did not change.

Due to the early stopping of the study, a difference in the composite endpoint between the groups was not expected. Our study, however, showed the intervention to be feasible and safe, in both academic and non-academic settings. Severe adverse events were reported in accordance with the guidelines of the Medical Device Regulation (MDR). Future adequately powered prospective trials using the newly developed investigational device are necessary to further determine the effects of CPPF in this patient population. Study protocols should include clearly defined endpoints and standardized definitions of clinical outcomes and complications. Premature study termination should be avoided to ensure robust and generalizable results. Nevertheless, our study confirms the findings of previous studies, namely that CPPF is associated with less postoperative blood loss.

Traditional methods to improve chest tube clearance such as milking and stripping of chest tubes are controversial. No clinical advantages of chest tube manipulation have been demonstrated compared to no manipulation. In addition, these methods can even be harmful by exerting very low negative intrathoracic pressures and thereby increasing the probability of tissue damage [19,20]. Other techniques to improve pericardial drainage have been studied before. Active tube clearance is a technique that aims to promote chest tube clearance by breaking clots mechanically in the lumen of the chest tubes [21]. Studies of active tube clearance show conflicting results with regard to a reduction in re-explorations for bleeding. Previous studies demonstrated promising results of active tube clearance for bleeding-related complications; however, a recent prospective study did not find any difference for active tube clearance compared to standard care [10,12,13].

Posterior pericardial drainage is a strategy that can refer to a posterior pericardiotomy, a procedure which involves creating an additional incision in the posterior pericardium at the end of the surgical procedure. Posterior pericardial drainage can also refer to the insertion of a chest tube in the posterior pericardium or a combination of the two mentioned techniques. Both techniques aim to facilitate the drainage of blood from the pericardial space. This strategy has been shown to be safe, with a significant reduction in cardiac tamponade, pericardial effusion, and postoperative atrial fibrillation [22].

These techniques demonstrate promising results. However, they focus on a single aspect only, either the patency of the chest tubes or the promotion of drainage from the pericardial space. The strength of CPPF is that it is an integrated approach. Our technique enhances the patency of chest tubes through continuous flushing and actively dilutes residual blood and clots in the pericardial space. In addition, flushing of the pericardial space is hypothesized to reduce fibrinolytic activity, as is frequently observed during negative surgical re-exploration. Bleeding-related complications are frequently observed in patients following cardiac surgery and are associated with adverse outcomes. The accumulation of excessive blood and clots in the mediastinum can lead to acute mechanical compression of the heart and lungs, resulting in a potentially life-threatening condition [9]. Subsequent medical interventions are often required to evacuate blood from the mediastinum and to stabilize the patient. Surgical re-exploration poses a great risk for patients, as it is associated with prolonged hospitalization and increased mortality. In addition, less invasive medical interventions, such as blood transfusions, are often required for stabilization purposes. However, the transfusion of blood products carries a significant financial burden and can potentially be harmful for the patient [2,3,5,23,24,25].

Comparison of blood loss between the CPPF and control groups posed an important methodological challenge. Because the intervention involved rinsing of the pericardial space with fluid, blood was inevitably mixed with irrigation fluid, rendering precise direct measurements of blood loss infeasible. Accordingly, and consistently with prior CPPF studies, blood loss was calculated rather than directly measured during the first 8 postoperative hours. In addition, a proportion of the irrigation fluid may remain intrathoracically and/or be absorbed, thereby reducing early drain output without necessarily reappearing in the drains, which further complicates quantification. These factors may have led to an underestimation of true blood loss in the CPPF group. Therefore, the observed reduction in early postoperative blood loss should be interpreted with caution and should not be overinterpreted. At present, no validated alternative method is available for more accurate quantification in this setting. Importantly, this limitation is unlikely to have affected the primary or other secondary endpoints, but it specifically constrains conclusions regarding blood loss.

There are further limitations to this study. The study was stopped prematurely due to the development of a new and substantially improved version of the investigational device. Continuing the study without implementation of this improvement was deemed ethically inappropriate. As a result of the early stopping, the study lacked sufficient power to assess the impact of CPPF on major clinical outcomes. It also precluded matched-group analyses. Despite these limitations, we believe it is important to report the available data, as the findings may still provide valuable insights and contribute to the existing body of evidence.

A relevant practical limitation of this study is that a relatively high proportion of patients randomized to CPPF did not ultimately receive the intervention due to technical or logistical constraints. These included issues related to device setup, the need for substantial manual handling, workflow integration in the operating room, and the availability of adequately trained personnel. This highlights that, in its current form, the implementation of CPPF is operationally demanding and may pose challenges even in a controlled trial setting. Importantly, such barriers may be equal or more pronounced in routine clinical practice, where time pressure, staffing constraints, and variability in local logistics are greater. Therefore, these findings underscore that feasibility and real-world applicability are important considerations and may limit widespread adoption unless procedural complexity and logistical requirements can be further reduced. While newer device iterations may improve usability, the present results should be interpreted in light of these practical implementation challenges.

Furthermore, it is essential to acknowledge that this study lacked blinding, which introduced a potential source of bias. This concern is particularly pertinent given that the primary endpoint, postoperative re-explorations for cardiac tamponade or non-surgical excessive bleeding, is inherently subjective. In an effort to mitigate bias, a clinical event committee was established to adjudicate instances of tamponade or excessive bleeding, distinguishing between surgical and non-surgical causes. However, it is imperative to note that the decision to undertake a re-thoracotomy was always made by the attending surgeon, who was not blinded to the randomization status.

In conclusion, this prematurely stopped, underpowered study did not show a reduction in postoperative re-exploration with CPPF. Further well-powered trials remain needed to determine whether CPPF impacts outcomes for cardiac surgery patients.

## Figures and Tables

**Table 1:** Patient demographics and baseline characteristics.

	CPPF (*n* = 79)	Control (*n* = 85)	*p*
age, years	68 [61–73]	66 [59–73]	0.73
sex, male	62 (78.5%)	74 (87.1%)	0.21
BMI, kg/m^2^	26.6 [24.1–30.2]	26.4 [24.0–29.7]	0.77
Euroscore II	1.73 [0.99–2.91]	1.49 [0.96–2.91]	0.46
cardiovascular risk factors			
smoking status			0.26
current	11 (14.5%)	21 (24.7%)	
former	31 (40.8%)	30 (35.3%)	
hypertension	42 (55.3%)	46 (56.1%)	1.00
hypercholesterolemia	33 (45.2%)	37 (44.6%)	1.00
diabetes mellitus	17 (21.5%)	23 (27.1%)	0.52
myocardial infarction	37 (46.8%)	43 (50.6%)	0.75
atrial fibrillation	11 (13.95%)	13 (15.3%)	0.98
comorbidities			
micro- or macrovascular disease	23 (29.5%)	19 (22.4%)	0.39
decompensation cordis	8 (10.3%)	8 (9.5%)	1.00
chronic pulmonary diseases	8 (10.1%)	10 (11.8%)	0.93
neurological disease	4 (5.1%)	10 (11.9%)	0.20
renal disease	9 (11.4%)	5 (5.9%)	0.32
malignancy	7 (9.0%)	5 (5.9%)	0.65
current infection	8 (10.1%)	4 (4.7%)	0.30
left ventricular function			0.54
good	40 (64.5%)	40 (58.0%)	
moderate	18 (29.0%)	26 (37.7%)	
poor	4 (6.5%)	3 (4.3%)	
coagulation status			
number of patients with preoperative anticoagulation use	61 (77.2%)	65 (76.5%)	1.00
type of anticoagulant use *			0.20
vitamin K antagonists	4 (5.9%)	0 (0%)	
heparins	3 (4.4%)	6 (8.2%)	
direct oral anticoagulants	5 (7.3%)	9 (12.3%)	
platelet aggregation inhibitors	53 (77.9%)	53 (72.6%)	
number of anticoagulants per patient			0.65
1	33 (54.1%)	41 (63.1%)	
2	19 (31.1%)	14 (21.5%)	
3	7 (11.4%)	8 (12.3%)	
4	2 (3.3%)	2 (3.1%)	
platelet count (·10^9^/L)	235 [220–301]	245 [200–309]	0.56
INR	1.00 [1.00–1.10]	1.00 [1.00–1.03]	0.57

Data are medians [IQR] or numbers (%); *, the total exceeds the number of patients as they could use more than one type of anticoagulant. Abbreviations: BMI, body mass index; CPPF, continuous postoperative pericardial flushing; INR, international normalized ratio.

**Table 2:** Intraoperative characteristics.

	CPPF (*n* = 79)	Control (*n* = 85)	*p*
type of surgery			0.40
CABG	50 (63%)	52 (61%)	
valve surgery	10 (13%)	6 (7%)	
CABG + valve surgery	4 (5%)	9 (11%)	
aortic surgery	8 (10%)	14 (16%)	
other	7 (9%)	4 (5%)	
surgery characteristics			
operation duration, min.	235 [201–304]	245 [203–300]	0.79
CPB duration, min.	111 [85–161]	104 [78–152]	0.66
cross-clamp duration, min.	73 [54–108]	75 [56–111]	0.70
surgically opened pleural space			0.56
none	25 (32%)	34 (40%)	
one	35 (44%)	30 (35%)	
two	19 (24%)	21 (25%)	
transfusion			
cell saver blood, mL	450 [225–691]	356 [125–538]	0.11
patients receiving any blood product	32 (41%)	27 (32%)	0.32
patients receiving PRBC	21 (66%)	16 (59%)	0.82
patients receiving FFP	8 (25%)	4 (15%)	0.52
patients receiving platelets	21 (66%)	20 (74%)	0.68
laboratory results at end of surgery			
hemoglobin, mmol/L	6.7 [6.0–7.3]	6.7 [6.1–7.2]	0.78
aPTT, sec.	28.3 [26.3–31.7]	27.7 [25.6–30.7]	0.47
PT, sec.	15.7 [14.7–16.7]	15.0 [14.0–16.0]	0.09

Data are medians [IQR] or numbers (%). Abbreviations: aPTT, activated partial thromboplastin time; CABG, coronary artery bypass grafting; CPB, cardiopulmonary bypass; CPPF, continuous postoperative pericardial flushing; FFP, fresh frozen plasma; PRBC, packed red blood cells; PT, prothrombin time.

**Table 3:** Re-thoracotomies.

	CPPF (*n* = 79)	Control (*n* = 85)	Risk Ratio (95% Confidence Interval)	*p*
**Primary endpoint**				
re-thoracotomy for				
tamponade or excessive bleeding due to non-surgical cause	3/79 (3.8%)	2/85 (2.4%)	1.61 (0.28–9.41)	0.67
**Secondary endpoints**				
components of the primary endpoint *				
for tamponade	2/79 (2.5%)	1/85 (1.2%)	2.15 (0.20–23.27)	0.61
for excessive bleeding due to non-surgical cause	1/79 (1.3%)	2/85 (2.4%)	0.54 (0.05–5.82)	1.00
re-thoracotomy for excessive bleeding due to surgical cause	1/79 (1.3%)	3/85 (3.5%)	0.36 (0.04–3.38)	0.62
re-thoracotomy not bleeding-related	3/79 (3.8%)	0/85 (0%)	∞	0.11

Data are numbers (%); *, re-thoracotomy could have been performed in patients for both reasons. Abbreviations: CPPF, continuous postoperative pericardial flushing.

**Table 4:** Other endpoints and patient follow-up.

	CPPF (*n* = 79)		Control (*n* = 85)	*p*
**Other secondary endpoints**				
blood loss *				
blood loss at 8 h, mL	270 [40–490]		355 [250–555]	0.02
number of patients with blood loss > 0.5 L at 8 h	15/67 (22.4%)		34/80 (42.5%)	0.014
number of patients with blood loss > 1 L at 8 h	7/67 (10.4%)		6/80 (7.5%)	0.57
number of patients who had a transfusion	14/79 (17.7%)		20/85 (23.5%)	0.36
patients receiving PRBC	13/79 (16.5%)		15/85 (17.6%)	1.00
PRBC, units **	2.0 [2.0–6.0]		2.0 [1.5–4.0]	0.56
patients receiving FFP	6/79 (7.6%)		8/85 (9.4%)	0.78
FFP, units **	2.5 [1.3–4.5]		2.5 [2.0–4.0]	0.95
patients receiving platelets	4/79 (5.1%)		11/85 (12.9%)	0.11
platelets, units **	1.5 [1.0–2.8]		1.0 [1.0–2.0]	0.60
number of patients with new-onset atrial fibrillation requiring treatment	19/79 (24.1%)		21/85 (24.7%)	1.00
electric cardioversion	0/19 (0%)		0/21 (0%)	
chemical cardioversion	17/19 (89.5%)		19/21 (90.5%)	
electric and chemical cardioversion	2/19 (10.5%)		2/21 (9.5%)	
number of patients with an intervention for fluid accumulation				
intervention for pericardial effusion	3/79 (3.8%)		8/85 (9.4%)	0.21
intervention for pleural effusion	0/79 (0%)		0/85 (0%)	1.00
**Patient follow-up**				
hospital length of stay, days	6.0 [4.0–9.0]		5.0 [4.0–8.0]	0.79
mortality rates				
ICU mortality	2/79 (2.5%)		1/85 (1.2%)	0.45
hospital mortality	2/79 (2.5%)		1/85 (1.2%)	0.45
30-day mortality	2/79 (2.5%)		1/85 (1.2%)	0.45
90-day mortality	2/79 (2.5%)		1/85 (1.2%)	0.45

Data are medians [IQR] or numbers (%); *, data are missing for 12 CPPF patients and for 5 control patients; **, in patients receiving PRBC, FFP or platelets. Abbreviations: CPPF, continuous postoperative pericardial flushing; FFP, fresh frozen plasma; ICU, intensive care unit; PRBC, packed red blood cells.

## Data Availability

Data collected in this study, study materials, and analytical methods will be made available to other researchers upon reasonable request for reproduction purposes.

## Supplementary Materials

The following supporting information can be downloaded at https://www.mdpi.com/article/10.3390/jcm15062151/s1, Figure S1: Investigational Device, Table S1: Per-protocol analysis of primary endpoint. Table S2: Posthoc analysis in high-risk patients, Statistical Analysis Plan, Research Protocol.

## Abbreviations

The following abbreviations are used in this manuscript: Amsterdam UMC = Amsterdam University Medical Center, Amsterdam, The Netherlands; CABG = coronary artery bypass grafting; CI = confidence interval; CONSORT = CONsolidated Standards Of Reporting Trials; CPPF = continuous postoperative pericardial flushing; DAPT = dual antiplatelet therapy; DHCA = deep hypothermic circulatory arrest; CPB = cardiopulmonary bypass; FLUID = FLUshing with Investigational Device; ICU = intensive care unit; IQR = interquartile range; LOCF = last observation carried forward; NaCl = sodium chloride; POAF = postoperative atrial fibrillation; SD = standard deviation.
