# Supplementary material for: Effects of Continuous Postoperative Pericardial FLUshing with Investigational Device on Postoperative Re-Explorations for Bleeding (FLUID)—Randomized Clinical Trial
Source: J Clin Med. 2026 Mar 11;15(6):2151. doi: 10.3390/jcm15062151 (PMC13026474; doi:10.3390/jcm15062151)
Supplement: Supplementary file 1 [file jcm-15-02151-s001.zip › jcm-4113872-supplementary/Research protocol - FLUID study - 04012026.pdf]

DocuSign Envelope ID: E42CD872-B126-44E1-9888-9E11235E4D76

CONTINUOUS POSTOPERATIVE PERICARDIAL FLUSHING AFTER GENERAL CARDIAC SURGERY PROCEDURES, WITH THE HAERMONICS INVESTIGATIONAL DEVICE : STUDY PROTOCOL OF THE FLUID (FLUsh with Investigational Device) TRIAL

**Protocol ID**	**NL74428 -FLUID**
**Version**	V 4.0
**Date**	24-10-2022
**Coordinating investigator /project leader**	Vacancy<br>Ad interim: prof. dr. R.J.M. Klautz<br>Professor of Cardiothoracic Surgery<br>AMC Medical Research Department, Amsterdam University Medical Centers, loc. AMC<br>Meibergdreef 9 1105 AZ Amsterdam, The Netherlands tel.: +31(0)6-26466089
**Principal investigator**	prof. dr. R.J.M. Klautz<br>Professor of Cardiothoracic Surgery<br>Dep. Cardiothoracic Surgery, Amsterdam University Medical Centers, loc. AMC<br>Meibergdreef 9, 1105 AZ, Amsterdam, The Netherlands<br>tel.: +31(0)20-5669111<br><br>A. De Weger<br>Dept. of Cardio-thoracic Surgery<br>Leiden University Medical Center<br>Albinusdreef 2, 2333 ZA, Leiden, The Netherlands<br>Tel.: +31(0)71-5269111<br><br>F.N. Hofman<br>St. Antonius Hospital<br>Koekoekslaan 1, 3435 CM, Nieuwegein, The Netherlands<br>Tel: +31 (0) 88 320 30 00<br><br>K.Lam<br>Catharina Hospital<br>Michelangelolaan 2, 5623 EJ, Eindhoven, The Netherlands<br>Tel: +31(0)40-2398680
**Sponsor**	Amsterdam University Medical Centers, loc. AMC<br>Meibergdreef 9, 1105 AZ, Amsterdam, The Netherlands<br>tel.: +31(0)20-5669111

DocuSign Envelope ID: E42CD872-B126-44E1-9888-9E11235E4D76

**Subsidising party**	Kansen voor West, an EFRO subsidy and Haermonics b.v.
**Independent expert (s)**	Dr. A. van Wijk, Congenital Cardiothoracic Surgeon, University Medical Center Utrecht, Wilhelmina Kinder Ziekenhuis (WKZ)<br>Lundlaan 6, 3584 EA Utrecht<br>tel.: +31 (0)6-42727223

PROTOCOL SIGNATURE SHEET

Name	Signature	Date
**Sponsor or legal representative:**<br>Amsterdam University Medical Centers, loc. AMC<br>Meibergdreef 9, 1105 AZ, Amsterdam<br>tel.: +31(0)20-5669111<br><br>**Head of Department:**<br>prof. dr. R.J.M. Klautz<br>Professor of Cardiothoracic Surgery	DocuSigned by:<br>Prof. Dr. R.J.M. Klautz<br>4001C67DD8164A9...	10/24/2022

TABLE OF CONTENTS

1. INTRODUCTION AND RATIONALE 9
2. OBJECTIVES 11
3. STUDY DESIGN 12
4. STUDY POPULATION 13
  4.1 Population (base) 13
  4.2 Inclusion criteria 13
  4.3 Exclusion criteria 13
  4.4 Sample size calculation 13
5. TREATMENT OF SUBJECTS 15
  5.1 Investigational product/treatment 15
6. INVESTIGATIONAL PRODUCT 17
  6.1 Name and description of investigational product 17
  6.2 Summary of findings from non-clinical studies 17
  6.3 Summary of findings from clinical studies 18
  6.4 Summary of known and potential risks and benefits 18
  6.5 Preparation and labelling of Investigational Medicinal Product 19
7. METHODS 20
  7.1 Study parameters/endpoints 20
    7.1.1 Main study parameter/endpoint 20
    7.1.2 Secondary study parameters/endpoints 20
    7.1.3 Other study parameters 21
  7.2 Randomisation, blinding and treatment allocation 21
  7.3 Study procedure 21
    7.3.1 Patient characteristics 21
    7.3.2 Blood sampling 22
    7.3.3 Imaging 22
    7.3.4 Cost-effectiveness 22
  7.4 Withdrawal of individual subjects 23
  7.5 Replacement of individual subjects after withdrawal 23
  7.6 Follow-up of subjects withdrawn from treatment 23
  7.7 Premature termination of the study 23
  7.8 Trial management during the COVID-19 pandemic 23
8. SAFETY REPORTING 25
  8.1 Temporary halt for reasons of subject safety 25
  8.2 AEs, SAEs and SUSARs 25
    8.2.1 Adverse events (AEs) 25
    8.2.2 Serious adverse events (SAEs) 25
    8.2.3 Serious Adverse Device Effects (SADE's) 25
    8.2.4 Investigator Responsibility 26
  8.3 Annual safety report 26
  8.4 Follow-up of adverse events 27

DocuSign Envelope ID: E42CD872-B126-44E1-9888-9E11235E4D76

8.5 Data Safety Monitoring Board (DSMB) 27
8.5.1 Specific issues 29
9. STATISTICAL ANALYSIS 31
9.1 Primary study parameter(s) 31
9.2 Secondary study parameter(s) 31
9.3 Other study parameters 32
9.4 Interim analysis 33
10. ETHICAL CONSIDERATIONS 34
10.1 Regulation statement 34
10.2 Recruitment and consent 34
10.3 Benefits and risks assessment, group relatedness 34
10.4 Compensation for injury 34
10.5 Incentives 34
11. ADMINISTRATIVE ASPECTS, MONITORING AND PUBLICATION 35
11.1 Handling and storage of data and documents 35
11.2 Monitoring and Quality Assurance 35
11.3 Amendments 35
11.4 Annual progress report 35
11.5 Temporary halt and (prematurely) end of study report 35
11.6 Public disclosure and publication policy 36
12. STRUCTURED RISK ANALYSIS 37
12.1 Potential issues of concern 37
12.2 Synthesis 37
13. REFERENCES 41
14. APPENDIX 44

DocuSign Envelope ID: E42CD872-B126-44E1-9888-9E11235E4D76

LIST OF ABBREVIATIONS AND RELEVANT DEFINITIONS

**ABR**	**General Assessment and Registration form (ABR form), the application form that is required for submission to the accredited Ethics Committee; in Dutch: Algemeen Beoordelings- en Registratieformulier (ABR-formulier)**
**ACT**	**Activated clotting time**
**AE**	**Adverse Event**
**AF**	**Atrial Fibrillation**
**aIE**	**acute Infective Endocarditis**
**APTT**	**Activated Partial Thromboplastin Time**
**AR**	**Adverse Reaction**
**CA**	**Competent Authority**
**CABG**	**coronary artery bypass grafting**
**CCMO**	**Central Committee on Research Involving Human Subjects; in Dutch: Centrale Commissie Mensgebonden Onderzoek**
**CI**	**confidence interval**
**CKMB**	**Creatine Kinase Muscle and Brain**
**COVID-19**	**COrona Virus Disease 2019**
**CPB**	**cardiopulmonary bypass**
**CRP**	**C- Reactive Protein**
**CV**	**Curriculum Vitae**
**CPPF**	**Continuous Postoperative Pericardial Flushing**
**DAPT**	**continued dual antiplatelet therapy**
**DHCA**	**Deep Hypothermic Circulatory Arrest**
**DSMB**	**Data Safety Monitoring Board**
**eCRF**	**Electronic Clinical Research Form**
**ECG**	**Electrocardiography**
**ECV**	**Electrocardioversion**
**EU**	**European Union**
**EudraCT**	**European drug regulatory affairs Clinical Trials**
**GCP**	**Good Clinical Practice**
**GDPR**	**General Data Protection Regulation; in Dutch: Algemene Verordening Gegevensbescherming (AVG)**
**hct**	**haematocrit**
**IC**	**Informed Consent**
**ICU**	**Intensive Care Unit**
**IL**	**InterLeukins**
**INR**	**InterNational Rate**
**IMDD**	**Investigational Medicinal Device Dossier**
**METC**	**Medical research ethics committee (MREC); in Dutch: medisch-ethische toetsingscommissie (METC)**
**MCTD**	**Mediastinal Chest Tube Drainage**

DocuSign Envelope ID: E42CD872-B126-44E1-9888-9E11235E4D76

**MDR**	**Medical Device Regulation**
**NZA**	**Dutch Healthcare Authority; in Dutch: nederlandse zorg autoriteit**
**POAF**	**Post-Operative Atrial Fibrillation**
**PTT**	**Partial Thromboplastin Time**
**(S)AE**	**(Serious) Adverse Event**
**SPC**	**Summary of Product Characteristics; in Dutch: officiële productinformatie IB1-tekst**
**Sponsor**	**The sponsor is the party that commissions the organisation or performance of the research, for example a pharmaceutical company, academic hospital, scientific organisation or investigator. A party that provides funding for a study but does not commission it is not regarded as the sponsor, but referred to as a subsidising party.**
**SUSAR**	**Suspected Unexpected Serious Adverse Reaction**
**UAVG**	**Dutch Act on Implementation of the General Data Protection Regulation; in Dutch: Uitvoeringswet AVG**
**WMO**	**Medical Research Involving Human Subjects Act; in Dutch: Wet Medisch-wetenschappelijk Onderzoek met Mensen**

DocuSign Envelope ID: E42CD872-B126-44E1-9888-9E11235E4D76

SUMMARY

Rationale: In two randomized clinical trials we have demonstrated that continuous postoperative pericardial flushing (CPPF) therapy can reduce postoperative blood loss and bleeding-related complications after cardiac surgery and that CPPF therapy is safe and feasible in an experimental setting.(1, 2) The Haermonics investigational device is a novel medical device that enables CPPF therapy to be used in daily clinical setting. The aim of this study is three-fold. First, to evaluate the safety and functionality of the Haermonics investigational device. Secondly, to investigate the effect of CPPF therapy on bleeding related complications in the adult cardiac surgery population. Thirdly, to explore the effect of CPPF therapy on intraluminal chest tube clogging.

Objective: The primary objective of this study is to assess the effects of CPPF, executed by the Haermonics investigational device, in comparison with standard care on clinically relevant endpoints, like re-explorations for either bleeding and/or cardiac tamponade, in a population of adult cardiac surgery patients . Secondary objectives are to assess the safety and feasibility of the Haermonics investigational device, to validate the haematocrit (hct)-sensor and pressure sensor, to investigate the effect of CPPF on blood loss and the cost-effectiveness of CPPF executed by the Haermonics investigational device compared to standard care.

Study design: This is a prospective, multicenter, open label, adaptive randomized clinical trial.

Study population: In an adult population, 992 patients scheduled for a general cardiothoracic surgery procedure with the use of cardiopulmonary bypass are randomized.

Intervention: One group receives CPPF therapy (inflow of 500 ml NaCl 0,9% flushing fluid into the pericardial cavity during the first 8 postoperative hours) executed with the Haermonics investigational device and the other group receives standard care.

Main study parameters/endpoints: The main study endpoint is the incidence of re-exploration for either cardiac tamponade and/or excessive bleeding due to non-surgical bleeding within a week after the cardiac surgery procedure.

Nature and extent of the burden and risks associated with participation, benefit and group relatedness: The development of expected adverse events, infection and fluid retention (in pleural and pericardial cavities), will be closely monitored via several imaging techniques and laboratory measurements. Study participants will be asked to fill out EQ-5D questionnaires at three timepoints, pre-operatively, 3 days postoperative and 3 months postoperative.

DocuSign Envelope ID: E42CD872-B126-44E1-9888-9E11235E4D76

1. INTRODUCTION AND RATIONALE

CPPF therapy

Prolonged or excessive bleeding after cardiac surgery can lead to a broad spectrum of secondary complications.(3-5) One of the underlying causes is incomplete wound drainage, with subsequent accumulation of blood and clots in the pericardium. It has been demonstrated that this retained blood and clots lead to even more fibrinolytic activity in the mediastinum and pericardial space, and therefore may contribute to increased or prolonged bleeding(6-8). Based on this principle, the method of continuous postoperative pericardial flushing (CPPF) has been invented and further developed. The hypothesis is that CPPF therapy works by mechanical cleaning properties and by diminishing fibrinolysis and inflammation. The CPPF protocol includes the inflow of NaCl 0,9% flushing fluid into the pericardial cavity during the first postoperative hours in patients who underwent cardiac surgery. In this way, the blood and clot mixture can be diluted into a lower viscosity solution, thereby enhancing the evacuation of blood and clots from the pericardial space and preventing chest tube obstruction.

The Haermonics investigational device

Because CPPF therapy includes the dilution of the normal postoperative mediastinal chest tube drainage (MCTD), the clinical assessment of the exact amount of blood loss is more difficult. Yet, blood loss is an important factor in clinical decision making, namely the decision if the patient needs a surgical re-exploration for postoperative bleeding or not. Roughly, in patients who receive CPPF therapy, blood loss can be estimated by extracting the total inflow flushing volume from the total MCTD. This method was used in the experimental setting of the previous CPPF trails but is considered unsuitable for use in daily practice because of three reasons. First, the required registration of in- and outflow volume is labour intensive. Secondly, because this registration can only be done intermittently, which can be dangerous in case of a fast bleeding rate. Thirdly, blood loss calculation could potentially be inaccurate because sometimes, clinically insignificant, amounts of flushing fluid are retained or absorbed in the pericardial or pleural spaces, thereby making the blood loss calculation inaccurate.

The first commercial Haermonics device will have four essential functionalities that make CPPF therapy safe and feasible for daily clinical use. 1) Automatic monitoring of the outflow volume, 2) Quantification of the content of the outflow volume by means of real time and continuous haematocrit (hct) analysis of the MCTD, 3) Warming of the flushing fluid to body temperature and temperature measurements of the flushing fluid, and 4) Continuous intrapericardial pressure measurement. The investigational device that will be used in this study will have all these functionalities, but available data will not be used for clinical decision making yet.

Previous studies

CPPF, executed with a researcher instead of a medical device, has been investigated in two randomized clinical trials (1, 2). The CPPF protocol included the inflow of 500 ml NaCl 0,9% flushing fluid into the pericardial cavity during the first 12 postoperative hours in patients who

DocuSign Envelope ID: E42CD872-B126-44E1-9888-9E11235E4D76

underwent cardiac surgery. In this way, the blood and clot mixture were diluted into a lower viscosity solution, thereby enhancing the evacuation of blood and clots from the pericardial space and preventing chest tube obstruction. In two distinct cardiac surgery populations, both trials showed CPPF led to a statistically significant reduction in the primary outcome, i.e., blood loss, while pooled data showed a statistically significant difference for the clinically most relevant secondary end points, like the incidence of re-interventions for either non-surgical bleeding and/or acute cardiac tamponade (0 vs. 8 in CPPF vs. control group).(2) The present study is powered to assess the effects of CPPF, executed by the Haermonics investigational device, in comparison with standard care on these clinically more relevant endpoints in a population of adult cardiac surgery patients.

DocuSign Envelope ID: E42CD872-B126-44E1-9888-9E11235E4D76

2. OBJECTIVES

Primary objective

To investigate the effects of CPPF, executed by the Haermonics investigational device, as compared to standard care on the incidence of re-exploration for cardiac tamponade and/or excessive bleeding due to non-surgical bleeding in adult patients, within a week after the cardiac surgery procedure.

Secondary objectives

Secondary objectives are to:

1. assess the safety and feasibility of a newly developed CPPF device, i.e., the Haermonics investigational device;
2. validate the haematocrit (hct) and pressure sensor of the Haermonics investigational device;
3. investigate the clinical effects of CPPF therapy on; blood loss, the use of blood products and administration of coagulation factors, new onset postoperative atrial fibrillation (POAF), and the length of stay at the intensive care unit (ICU) and hospital;
4. to explore the effect of CPPF therapy on intraluminal chest tube clogging;
5. to estimate cost-effectiveness of the CPPF therapy with the Haermonics investigational device by assessing cost per quality adjusted life year (QALY), calculated from the health utility gain scores obtained with the EuroQol-5D (EQ-5D) questionnaire. Cost will include direct medical costs, consisting of hospital days, ICU-days, medication, transfusion requirements, costs related with in-hospital complications.

DocuSign Envelope ID: E42CD872-B126-44E1-9888-9E11235E4D76

3. STUDY DESIGN

This is a prospective, multicenter, open label, adaptive, randomized clinical trial that will enroll patients scheduled for a general cardiothoracic surgery procedure with the use of cardiopulmonary bypass, in six to seven Dutch medical centers: the Amsterdam UMC, location AMC, Amsterdam, LUMC, Leiden, St. Antonius Hospital, Nieuwegein, Catharina Hospital, Eindhoven and 2 to 3 additional centers. We expect to include patients over a 12-month period, which may be extended depending on the promising zone sample size re-estimation that will be conducted after 75% of the required participants has completed the follow-up period of 1 week to assess the primary outcome. Study procedures are performed according to the planned time schedule in adherence to visit intervals indicated given in the SPIRIT diagram (appendix 1, Table 1).

Patients are excluded for this study if any of the following criteria apply: (see chapter 4.3 for details on inclusion and exclusion criteria)

- Euroscore II > 20%
- Intraoperatively diaphragm injury leading to an open connection between the thoracic and abdominal cavity
- Age <18 years
- Inability to understand study information
- Participation in any study involving an investigational drug or device
- Emergent procedures
- Procedures performed off pump without cardiopulmonary bypass
- Minimal invasive cardiac surgery procedures (e.g. minithoracotomy and hemisternotomy)

DocuSign Envelope ID: E42CD872-B126-44E1-9888-9E11235E4D76

4. STUDY POPULATION

4.1 Population (base)

Adults undergoing a general cardiothoracic surgery procedure with the use of cardiopulmonary bypass are eligible for participation.

4.2 Inclusion criteria

In order to be eligible to participate in this study, a subject should be scheduled for a general cardiothoracic surgery procedure with the use of cardiopulmonary bypass, amongst others, the main categories are;

- Coronary artery bypass grafting (CABG),
- Valve surgery,
- CABG combined with valve surgery
- Elective patients scheduled for aortic surgery (including valve sparing root replacement (VSRR), Bentall procedures, ascending aorta- aortic arch replacement)

Including the initial study population:

- Patients scheduled for CABG with continued DAPT
- Patients with aIE scheduled for valve replacement
- Patients scheduled for complex or multiple cardiac (redo) procedures with an (expected) CPB time >300 minutes
- Patients undergoing aortic surgery with DHCA

4.3 Exclusion criteria

A potential subject who meets any of the following criteria will be excluded from participation in this study:

- Euroscore II > 20%
- Intraoperatively diaphragm injury leading to an open connection between the thoracic and abdominal cavity
- Age < 18
- Inability to understand study information
- Participation in any study involving an investigational drug or device
- Emergent procedures
- Procedures performed off pump, without the use of cardiopulmonary bypass.
- Minimal invasive cardiac surgery procedures (e.g. minithoracotomy and hemisternotomy)

A register is kept of all patients evaluated for inclusion and reason(s) for exclusion.

4.4 Sample size calculation

Re-explorations for either cardiac tamponade and/or excessive bleeding due to non-surgical bleeding occur in 3.6% in the target population of our study according to scientific literature[16-18] and 5.0% according the Netherlands Heart Registry.[19] Pooled data of two pilot trials of CPPF and one completed randomized controlled trial on the effects of active

DocuSign Envelope ID: E42CD872-B126-44E1-9888-9E11235E4D76

tube clearance suggest reductions in re-exploration of bleeding of 72% to 100% can be achieved.[1,2,20] For the current study, we expect that CPPF with Haermonics Flush will be able to reduce the number of re-explorations for either cardiac tamponade and/or excessive bleeding due to non-surgical bleeding by 70%. To detect a reduction in the proportion of re-explorations from 4.5% with standard care to 1.35% with Haermonics Flush, with a Type I error 5% and power of 80%, and equal allocation to two arms, and a suspected drop out of 10% we would require the inclusion of 496 patients in each arm of the trial, i.e., 992 in total. A promising zone sample size re-estimation will be used to allow continuation of the study beyond the originally envisioned sample size to prevent the study from being underpowered after completion. This means that the required sample size may be increased to a sponsor's maximum of 1450 participants. A detailed description of this procedure is provided in section 8.5. Participating centers will not be informed of interim results, but instead will be instructed at the start of the study to recruit participants until informed to stop recruiting. This is important to prevent an increase in the risk of operational bias if the announcement of the sample size re-estimation conveys the anticipation of an effective treatment.

DocuSign Envelope ID: E42CD872-B126-44E1-9888-9E11235E4D76

5. TREATMENT OF SUBJECTS

5.1 Investigational product/treatment

Standard care

To validate the hct sensor under investigation (DATAmed en DEMCON) the accuracy of the measurement during analysis of whole blood, with a higher hct than CPPF diluted blood, has to be investigated. To gather data of whole blood analysis of the hct sensors under investigation, the Haermonics investigational device will be connected to the standard care outflow chest tube of patients in the control group. The inflow tract of the Haermonics investigational device will not be connected, so there will be no postoperative flushing of the pericardial cavity.

Patients randomized to standard care group receive one chest tube in the pericardial space and one chest tube in the anterior mediastinum, according to local standard protocols. Additionally, each surgically opened pleural cavity is drained separately. All chest tubes will be connected to each other with the use of a Y-piece. To enable hct measurements of the MCTD volume of patients in the standard care group the Haermonics investigational device will be connected to this single chest tube. The outflow drain will be elongated, a costume made buffer volume canister (Demcon, Eindhoven, The Netherlands) with 2 level sensors (Introtec PLD-L, Brussels, Belgium) and a syringe (Coviden, Monoject 140 CC, sterile, male luer lock) will be placed in the outflow tract. Two pinch valves (Clippart, NPV4-1O-07-24, Belgium), a custom made hct sensor (Demcon, Eindhoven, The Netherlands) and a second hct sensor (DataMED Obba, Milan Italy) will be placed around the elongated part of the outflow drain (for a visual, see appendix 2, figure 1). The pinch valves are controlled by the lower level sensor of the buffer volume holder, the first valve will open and the second valve will close if an amount of 50 ml blood is stored in the buffer volume holder. After the first valve opens and the second valve closes, the part of the outflow tract connected to the syringe will be filled with blood and the syringe will collect 30 ml blood and inject this exact volume into the part of the outflow tract where the hct sensors are placed. MCTD samples will be drawn from the outflow tube every hour during the first 10 hours after surgery. To facilitate this sampling a sample port will be added to the outflow tract. Finally, the outflow drain will drain into a standard care closed vacuum drain collection system (Redax® DRENTECH® Simple Plus, Poggio Rusco, Italy).

To ensure adequate drainage in the event of a (mechanical) obstruction in the outflow tract of the Haermonics investigational device, an extra outflow drain will be placed at the top of the buffer volume canister and connected to a standard care closed vacuum drain collection system under negative pressure of 15cmH2O.

CPPF group

In addition to the aforementioned, for patients randomized to the study group an extra infusion tube (Ch10 or Ch12 Redivac drain, Dispo Medical, Hattemerbroek, the Netherlands) will be inserted through an extra incision hole and positioned in the pericardial space. This extra infusion tube is directly connected to the bag of irrigation solution (NaCl 0,9%), the extra infusion tube will run through a volumetric pump (Volumed®µVP7000 by Acromed,

DocuSign Envelope ID: E42CD872-B126-44E1-9888-9E11235E4D76

Kloten, Swiss) and through a fluid heating device (Fluido Compact® fluid warmer by The Surgical Company, Amersfoort, the Netherlands). A pressure sensor (Truwave, Edwards, Newbury, United Kingdom) will be connected to the infusion line to measure the intrapericardial pressure by monitoring the pressure in the infusion line which is constantly filled with NaCl. CPPF will be started at sternal closure and will be performed continuously at a fixed flow rate of 500 ml/hour, until the total irrigation volume of 4000ml has been infused.

DocuSign Envelope ID: E42CD872-B126-44E1-9888-9E11235E4D76

6. INVESTIGATIONAL PRODUCT

6.1 Name and description of investigational product

Name

The Haermonics investigational device

Device description

The device will have three essential functionalities: 1) Automatic monitoring of the outflow volume, 2) Quantification of the content of the outflow volume by means of real time and continuous haematocrit (hct) analysis of the MCTD, 3) Warming of the flushing fluid to body temperature, and 4) Continuous intrapericardial pressure measurement. The investigational device that will be used in this study will have all these functionalities, but available data will not be used for clinical decision making yet.

Intended purpose

The Haermonics Investigational device system is designed to flush the peri-surgical space (peri-cardial, pleural and mediastinal) after cardio-thoracic surgery with the intention to reduce post-operative bleeding and post-operative complications related to bleeding or coagulation. The system works by continuous flushing of the peri-surgical space via a drainage with a saline rinsing solution to enhance the evacuation of blood and clots in the pericardial cavity and mediastinum. The Haermonics system reduces clogging of the chest tubes, thereby maintaining patency of the drainage and preventing subsequent accumulation of blood and clots in the pericardial space. The Haermonics system supports the measurement of/monitors the post-operative blood loss and intra-cavital pressure. The product is intended to be inserted in open and/or minimally invasive surgical procedures under sterile conditions.

Intended user

The user of the Haermonics investigational device system should be a qualified operator. The operator should have knowledge of the system and data interpretation, obtained via medical education, system manuals and/or specific courses. He/she is the person operating the device.

6.2 Summary of findings from non-clinical studies

Earlier technical research and exploration work done by Haermonics in cooperation with Lifetec (Eindhoven, The Netherlands), showed that measurement of hct via non-invasive optical sensors in the drain is feasible with an accuracy of 7%. Commercial sensors for this measurement principle are available on the market, however the range of these sensors was (at that time, 2015-2016) not covering the application of Haermonics (blood/saline solution ranging from 100% blood to 100% saline). A detection range of 4% hct to 45% hct for the mixture was found to be clinically acceptable, and a sensor with this measurement range was developed and validated in lab settings with porcine blood.

In order to calculate the amount of blood in a mixture, the hct value measured needs to be

DocuSign Envelope ID: E42CD872-B126-44E1-9888-9E11235E4D76

correlated to an accurate volume to which that hct value applies. Volume measurement was conceptually investigated but no prototypes have been built until now in the Haermonics investigational device. From the earlier experiments at Lifetec it was suggested to use a discrete volume measurement principle that collects a certain volume sample size in the outflow. The Haermonics investigational device has adopted that concept.

6.3 Summary of findings from clinical studies

CPPF, executed with a researcher instead of a medical device, has been investigated in a pilot trial and two randomized clinical trials. (1, 2, 14) The CPPF protocol included the inflow of 500 ml NaCl 0,9% flushing fluid into the pericardial cavity during the first 12 postoperative hours in patients who underwent cardiac surgery. In two distinct cardiac surgery populations, both trials showed CPPF led to a statistically significant reduction in the primary outcome, i.e., blood loss. Comparable findings were obtained by Kara and Erden, who used a similar CPPF protocol to evaluate the safety and feasibility in a group of patients that underwent isolated CABG. They observed a reduction in mean blood loss of 257.24 ml (38%) in the CPPF group.(15) Pooled data of our two randomized trials showed significant differences for clinically important secondary endpoints like re-interventions for non-surgical bleeding or acute cardiac tamponade (CPPF groups 0 versus 8 in the standard care group, $p = 0.007$). Adverse events related to CPPF therapy were not observed in any of the CPPF clinical trials.

6.4 Summary of known and potential risks and benefits

The known benefits associated with the CPPF therapy follow from the previous studies mentioned above; reduction of blood loss and reinterventions for bleeding related complications. Potential benefits are:

1. reduction of the incidence or duration of postoperative atrial fibrillation
2. reduced intensive care and hospital stay
3. reduction in wound adhesions and scar tissue formation

CPPF group

There are no known risks related to the CPPF method, as described in previous trials. (1, 2, 14) Although the risk is very small, a potential risk of CPPF, executed by the investigational device, is an increased induction of cardiac tamponade while the flushing fluid is infused into the pericardial space. However, the risk of chest tube obstruction is minimized by the dilution of the blood and clot mixture and therefore the chance of tamponade is lower than with current drainage systems. Moreover, cardiac tamponades have not been observed in patients randomized to the CPPF group during previous studies, although theoretically an obstruction or kinking of one or more of the outflow chest tubes can never be excluded. Therefore, flow rate adjustments are made in the event of fluid accumulation and partial or total drain obstruction. In the event of > 200 ml fluid accumulation in one hour for a patient with closed pleural cavities, > 300ml for a patient with one opened pleural cavity and > 400ml for a patient with two opened pleural cavities, the flow rate will be lowered to 100mL/hour and if not evacuated during the next two hours, CPPF will be stopped. In the event of total

DocuSign Envelope ID: E42CD872-B126-44E1-9888-9E11235E4D76

thoracic drain/ outflow tract obstruction CPPF will be stopped until the obstructed chest tube(s) are functional again. Other potential risks are (deep) sternal wound infections.

Standard care group

To gather data of whole blood analysis of the hct sensors, the Haermonics investigational device will be connected to the standard care outflow chest tube of patients in the control group. If a (mechanical) obstruction in the outflow tract of the Haermonics investigational device occurs, a cardiac tamponade could be induced. Therefore, a bypass from the buffer volume canister to a standard care closed vacuum drain collection system under negative pressure of 15cmH2O, is implemented in the investigational device. Thereby, ensuring adequate drainage in the event of a (mechanical) obstruction in the investigational device.

All previously described adverse events will be monitored by the DSMB and they will be recorded in the electronic Clinical Research File (eCRF) by the investigator when observed by the investigator or by the staff. Information about the DSMB can be found in section 8.5 and a full summary of adverse events are described in appendix L4 of the protocol. Additional hazard to the patient might be caused when one of the primary assessment methods (x-ray) for possible complications is used on the ICU.

6.5 Preparation and labelling of Investigational Medicinal Product

Preparation and labelling of the investigational device prior to use will be performed on the day of surgery. Outside the operating room (OR), the investigational device will be tested for full functionality by verifying that the battery is fully charged, and the infusion warming device and volumetric pump are properly working. All required disposables will be unpacked and installed; if the sterility is broken before use, all the disposables will be replaced. The investigational device will be connected to the mobile vacuum unit after which the system will be primed , then all sensors will be tested for full functionality. Inside the OR, the sterile part of the inflow tube and outflow drain will be passed to the scrub nurse to be connected to the inflow tube positioned in the pericardial space and the Y-piece on the chest tubes, respectively.

DocuSign Envelope ID: E42CD872-B126-44E1-9888-9E11235E4D76

7. METHODS

7.1 Study parameters/endpoints

7.1.1 Main study parameter/endpoint

The incidence of re-exploration for either cardiac tamponade and/or excessive bleeding due to non-surgical bleeding within one week after surgery.

7.1.2 Secondary study parameters/endpoints

To assess safety and feasibility of the Haermonics investigational device:

- adverse events as listed in appendix L4

To validate the sensors

hct-sensor:

- actual blood loss volume manually calculated by subtracting the total infused CPPF volume from the total MCTD volume compared with;
- hct values of the MCTD volume measured by the Haermonics investigational device (hourly during the first 10 hours in the ICU and at chest tube removal) compared with;
- hct values of MCTD samples (drawn from the outflow tube during the first 10 hours in the ICU and at chest tube removal) performed by the local laboratory.

- *pressure-sensor:*
    - Comparison of central venous pressure measurements and intrapericardial pressure measurements measured by the Haermonics investigational device.

To investigate the clinical effects:

- incidence of new onset postoperative atrial fibrillation requiring medical therapy or electrocardioversion (ECV) within 30 days after the cardiac surgery procedure
- the use of blood products and administration of coagulation factors within 30 days after the cardiac surgery procedure
- ICU stay (hours) and hospital stay (days)

To explore the effect of CPPF on intraluminal chest tube clogging:

- Upon removal, the chest tubes will be cut transversely and a picture will be taken (provided a research student is available to take the picture). A single, dedicated investigator, blinded for treatment allocation, will classify the chest tube as patent, partially or completely obstructed.

To assess cost-effectiveness:

- costs of all procedures associated with the standard and the experimental intervention as described below (chapter 7.1.3)
- Quality of life (EQ-5D)

DocuSign Envelope ID: E42CD872-B126-44E1-9888-9E11235E4D76

7.1.3 Other study parameters

- PATIENT CHARACTERISTICS:
  - Sex
  - Year of birth
  - Cardiovascular risk factors
  - Co-morbidity
  - Surgical history
  - Medication
  - Logistic Euroscore
  - Length
  - Weight

- STANDARD DIAGNOSTIC:
  - ECG: sinusrhythm, AF, pacemaker rhythm, junctional rhythm
  - Lab: CRP, leukocytes, INR, platelets, haemoglobin, haematocrit, creatinine
  - X-ray: pericardial effusion,
  - Echo: left ventricle function, right ventricle function (Tapse), pericardial effusion

- OPERATIVE DATA:
  - Procedure: operation time, CPB time, cross-clamp time, DHC(A) time, type of procedure, redo surgery, type of valvular heart surgery, number of opened pleural cavities and chest tubes, transfusion requirements,
  - Lab (during surgery): haematocrit,

7.2 Randomisation, blinding and treatment allocation

Randomization occurs intraoperatively, once there is certainty that the patient could receive either intervention, by a research assistant using a password detected, web-based randomization tool (Castor®). Randomisation will be stratified by site and blocks of sizes 4, 6 and 8 will be used. Due to the nature of the intervention neither participants nor staff can be blinded to allocation, outcome assessors will be blinded to treatment allocation.

7.3 Study procedure

7.3.1 Patient characteristics

At the start of the study patient characteristics are determined:

- <u>General</u>
  - Sex
  - Year of birth
  - Risk factors (smoking, hypertension, hypercholesterolemia, diabetes, history of cardiac infarction)

DocuSign Envelope ID: E42CD872-B126-44E1-9888-9E11235E4D76

  - Co-morbidity (atrial fibrillation, micro-and macrovascular co-morbidity, decompensatio cordis, pulmonary disease, renal disorders, neurological disorders, malignancy and infections)
  - Surgical history
  - Medication (type, dosage, time)
  - Euroscore
  - Quality of life (EQ-5D)

- Physical examination
  - Weight (kg)
  - Length (cm)
  - Body Mass Index (kg/m2)

7.3.2 Blood sampling

Baseline samples will be obtained preoperative via venous puncture, concomitantly with routine sampling. Intra-and postoperative systemic blood samples will be drawn from the central venous line (jugular vein) and are all part of routine sampling, one (additional) haemoglobin analysis will be performed after 8 hours stay in the ICU. Postoperative pericardial blood samples will be drawn from the MCTD. Sampling will not interfere with the duration of the operation and ICU stay.

7.3.3 Imaging

Echocardiography is performed in all patients at baseline and postoperatively, according to standard protocols, except for some CABG patients, where TTE will be additional to standard care protocol. Echocardiography's are performed and analysed locally. Chest radiography is performed according to standard protocol in all patients at baseline, directly postoperatively and on the 2nd-7thday postoperatively to evaluate pleural effusion.

7.3.4 Cost-effectiveness

The costs of the standard care and of CPPF group will be compared. Only direct hospital costs will be included in the analysis, the time horizon will be according to the follow-up of the study, 3 months, therefore discounting of costs and is considered redundant. We will estimate the costs based on resource use (number of procedures) and unit costs. Hospital procedures will be collected from hospital databases on all procedures. These procedures will be linked to unit costs which will be calculated according to guidelines for economic evaluation in health care research, primarily by using the reimbursement prices issued by the Dutch Healthcare Authority (NZA), the Dutch manual or costing research in health care and for medication the Dutch formulary (www.medicijnkosten.nl). Costs of the reoperation will preferably be based on microcosting (Activity Based Costing Method). To assess the improvement of quality of life, all patients will be asked to fill out the EQ-5D questionnaire at baseline, 3 days postoperatively and 3 months postoperatively. On the third day postoperative some patient will still be admitted at the ICU, if patients are unable to fill out the questionnaire, a research team member will make an effort to assist the patient in filling out

DocuSign Envelope ID: E42CD872-B126-44E1-9888-9E11235E4D76

the questionnaire. EQ5D will be used to calculate utility scores and subsequently calculated the quality-adjusted life years (QALYs). QALYs will be calculated using an area under the curve approach.

7.4 Withdrawal of individual subjects

Subjects can leave the study at any time for any reason if they wish to do so without any consequences. The investigator or physician can decide to withdraw a subject from the study for urgent medical reasons.

7.5 Replacement of individual subjects after withdrawal

A patient who withdraws or is withdrawn prior to surgery will be replaced to obtain the required number of evaluable patients. Every effort will be made to collect all required data until the time of withdrawal.

7.6 Follow-up of subjects withdrawn from treatment

Patients withdrawn from treatment at any time after surgery will continue with standard care treatment and this does not constitute withdrawal of that patient from statistical analysis. Every effort will be made to collect all required data until the time of withdrawal. From the moment of withdrawal all follow-up data will be collected throughout the defined follow-up period.

7.7 Premature termination of the study

If there is unequivocal evidence of treatment benefit or harm this study will be prematurely terminated. Evaluation of both benefit and harm will take place during interim analysis. To account for the fact that data will be analysed multiple times during the course of the trial the Haybittle-Peto alpha spending function will be used to correct for any potential inflation of the type I error (ref met endnote). This means that, at interim analysis, a p-value < 0.001 will be considered statistically significant and will indicate a difference in either benefit or safety.

7.8 Trial management during the COVID-19 pandemic

Managing a multicenter clinical trial in the current climate of COVID-19 can be challenging. The current situation has necessitated timely problem solving to ensure that this trial remains open, and most importantly, that participant safety is monitored. Government directed restriction on the movement of people; risk of infection to trial participants and staff; requirement to self-isolate if ill, are some of the major challenges identified.

Recruitment

Our study population with pre-existing cardiovascular disease are likely to have comorbidities which are associated with poorer clinical outcomes in confirmed COVID-19 cases (ref met endnote). Despite this, the future consequences of delayed cardiac surgery often outweighs the risk of a COVID-19 infection for the study candidates. Therefore, it is expected that recruitment of new participants will develop as usual or at a slightly lower rate. To reduce the risk of COVID-19 infection to trial candidates and staff, study information can be provided by the research team member during a telephone clinical visit or by the clinician during a

standard care visit. The study information letter and informed consent form can be posted to candidates and returned by mail.

Data collection

Literature tells us that existing restrictions and difficulties adhering to trial protocols will lead to missing data and trial drop-outs due to difficulties in conducting follow-up appointments. (ref met endnote). However, face-to-face follow-up appointments are not required to gather study data since all information can be obtained from the electronic patient files from the study center or the referral hospital of the study candidate.

Training, communication and management of the research team

Correspondence between research team members, monitors, sponsors, labs and external companies will be via email. Training for the research team will be online via Skype, Zoom or MS Teams, if possible. An online telephone group for the research team, e.g. WhatsApp, will be established for troubleshooting and social support of the research team.

8. SAFETY REPORTING

8.1 Temporary halt for reasons of subject safety

In accordance to section 10, subsection 4, of the WMO, the sponsor will suspend the study if there is sufficient ground that continuation of the study will jeopardise subject health or safety. The sponsor will notify the accredited METC without undue delay of a temporary halt including the reason for such an action. The study will be suspended pending a further positive decision by the accredited METC. The investigator will take care that all subjects are kept informed.

8.2 AEs, SAEs and SUSARs

8.2.1 Adverse events (AEs)

Adverse events are defined as any undesirable experience occurring to a subject during the study, whether or not considered related to the investigational device or CPPF therapy. All adverse events reported spontaneously by the subject or observed by the investigator or his staff will be recorded.

8.2.2 Serious adverse events (SAEs)

A serious adverse event is any untoward medical occurrence or effect that

- results in death;
- is life threatening (at the time of the event);
- requires hospitalisation or prolongation of existing inpatients' hospitalisation;
- results in persistent or significant disability or incapacity;
- is a congenital anomaly or birth defect; or
- any other important medical event that did not result in any of the outcomes listed above due to medical or surgical intervention but could have been based upon appropriate judgement by the investigator.

For a full list of prespecified (serious) adverse event see appendix L4. An elective hospital admission will not be considered as a serious adverse event.
The investigator will report all SAEs to the sponsor without undue delay after obtaining knowledge of the events. The sponsor will report the SAEs through the web portal *ToetsingOnline* to the accredited METC that approved the protocol, within 7 days of first knowledge for SAEs that result in death or are life threatening followed by a period of maximum of 8 days to complete the initial preliminary report. All other SAEs will be reported within a period of maximum 15 days after the sponsor has first knowledge of the serious adverse events.

8.2.3 Serious Adverse Device Effects (SADE's)

Adverse reactions are all untoward and unintended responses to the Haermonics investigational device related to any dose administered.

Unexpected adverse reactions are SUDE's if the following three conditions are met:

DocuSign Envelope ID: E42CD872-B126-44E1-9888-9E11235E4D76

1. the event must be serious (see chapter 8.2.2);
2. there must be a certain degree of probability that the event is a harmful and an undesirable reaction to the product under investigation, regardless of the administered dose;
3. the adverse reaction must be unexpected, that is to say, the nature and severity of the adverse reaction are not in agreement with the product information as recorded in:
   - Investigational Medical Device Dosier (IMDD, appendix D2)

The sponsor will report expedited the following SADE's through the web portal *ToetsingOnline* to the METC:

- SADE's that have arisen in the clinical trial that was assessed by the METC;
- SADE's that have arisen in other clinical trials of the same sponsor and with the same medicinal product, and that could have consequences for the safety of the subjects involved in the clinical trial that was assessed by the METC.

The remaining SADE's are recorded in an overview list (line-listing) that will be submitted once every half year to the METC. This line-listing provides an overview of all SADE's from the study product, accompanied by a brief report highlighting the main points of concern.
The expedited reporting of SADE's through the web portal ToetsingOnline is sufficient as notification to the competent authority.

The expedited reporting will occur not later than 15 days after the sponsor has first knowledge of the adverse reactions. For fatal or life-threatening cases the term will be maximal 7 days for a preliminary report with another 8 days for completion of the report.

8.2.4 Investigator Responsibility

All participating study centres will appoint one researcher as responsible for the accurate documentation of all possible study-related adverse events SAEs and for reporting these events to the coordinating investigator.

8.3 Annual safety report

In addition to the expedited reporting of SUSARs, the sponsor will submit, once a year throughout the clinical trial, a safety report to the accredited METC.
This safety report consists of:

- a list of all suspected (unexpected or expected) serious adverse reactions, along with an aggregated summary table of all reported serious adverse reactions, ordered by organ system, per study;
- a report concerning the safety of the subjects, consisting of a complete safety analysis and an evaluation of the balance between the efficacy and the harmfulness of the product under investigation.

DocuSign Envelope ID: E42CD872-B126-44E1-9888-9E11235E4D76

8.4 Follow-up of adverse events

All Aes will be followed until they have abated, or until a stable situation has been reached. Depending on the event, follow up may require additional tests or medical procedures as indicated, and/or referral to the general physician or a medical specialist.

SAEs need to be reported till end of study within the Netherlands, as defined in the protocol.

8.5 Data Safety Monitoring Board (DSMB)

The study population has a high risk of serious complications, which are inherent to their vulnerable condition and unrelated the CPPF procedure or the Haermonics investigational device, which are under evaluation in this trial. Prespecified context specific SAEs are evaluated by a Data Safety Monitoring Board, consisting of the following members: prof. dr. Schultz (ICU), prof. dr. Levi (internal medicin), and dr. Steyerberg (statistics), that acts as an independent, expert and advisory monitor of participant safety.

After 75% of participants has completed the follow-up period of 1 week to assess the primary outcome of the study, a promising zone sample size re-estimation (SSR) will be performed by an independent statistician. Two input parameters of the original sample size calculation (see section 4.4) will be assessed at this interim analysis: 1) the incidence of the primary outcome in the control group, i.e., currently estimated to be 4.5%, and 2) the relative reduction in the incidence of the primary outcome in the intervention group as compared to the control group, i.e., currently estimated to be 70%. Based on observed values for both parameters at interim we defined three zones of a promising zone SSR (see Figure 2) [22:

- Favorable: The conditional power is at least 80% (green zone in Figure 2), meaning no sample size increase is needed to maintain power. The number of participants as determined in the original sample size calculation will be retained, so a total of 992 participants will be included.
- Promising: The conditional power is between 60% and 80% (orange zone in Figure 2), meaning the combination of the observed incidence of the primary outcome in the control group and the observed relative reduction in the intervention group is lower than expected, but assuming that both observed parameters exactly reflects the true values of these parameters and that these trends continue, the power can be recovered, by increasing the sample size. When a conditional power between 60% and 80% is observed at interim, the required sample size will be re-estimated using the observed incidence in the control group and the observed relative reduction in the intervention group such that a conditional power of 80% will be achieved at study completion. The sample size can be increased to a maximum as defined by the study's sponsor of 1450 participants
- Unfavorable: The conditional power is below 60% (red zone in Figure 2), meaning the combination of the observed incidence of the primary outcome in the control group and the observed relative reduction in the intervention group is lower than expected and not sufficient to warrant the increase in sample size needed to maintain study power. When a conditional power below 60% is observed at interim, an increase in sample size will not be considered. Instead, the number of participants as determined

DocuSign Envelope ID: E42CD872-B126-44E1-9888-9E11235E4D76

in the original sample size calculation will be retained, so a total of 992 participants will be included.

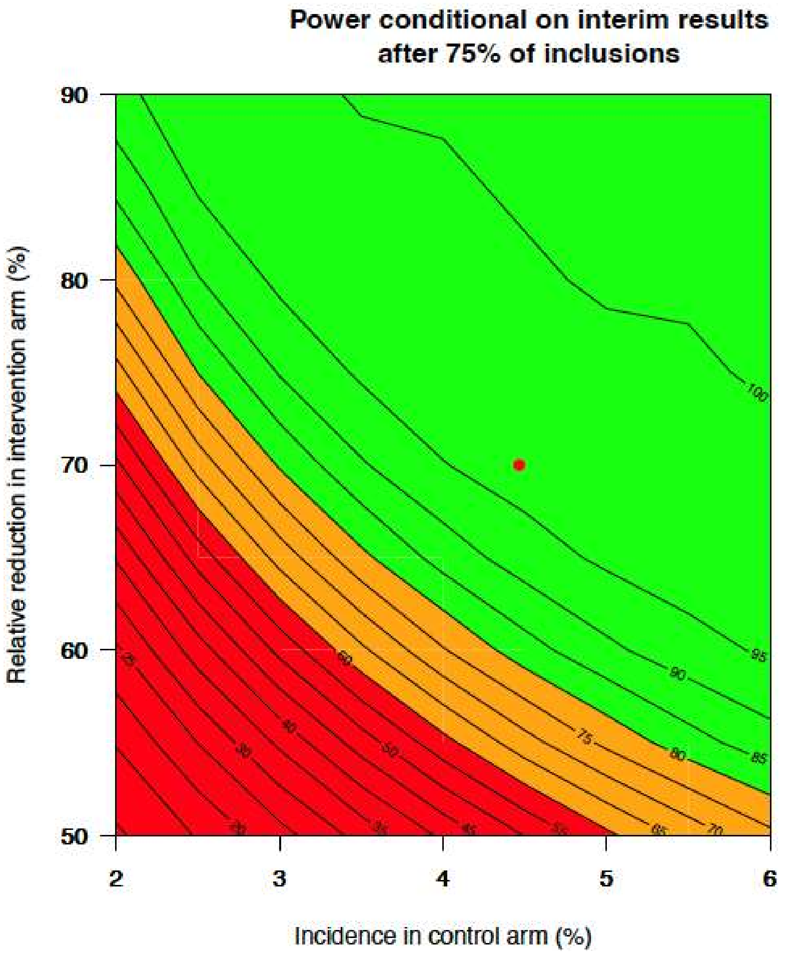

Figure 2: Conditional power based on a combination of the incidence of the primary outcome in the control arm and the relative reduction in the intervention arm at interim analysis. The contour lines indicate the conditional power. The green area indicates the favorable zone, the orange area the promising zone, and the red area the unfavorable zone. The red circle indicates the input parameters of the original sample size calculation, i.e., expected incidence in the control arm of 4.5% and an expected relative reduction in the intervention arm of 70%.

In addition to the promising zone sample size re-estimation, a formal interim analysis will be performed to assess efficacy and safety. To account for the fact that data will be analysed multiple times during the course of the trial the Haybittle-Peto alpha spending function will be used to correct for any potential inflation of the type I error. This means that, at interim analysis, a p-value < 0.001 will be considered statistically significant and will indicate a difference in either benefit or safety. The type I error of the final analysis of the trial will not be affected by the promising zone sample size re-estimation and is further accounted for by using the Haybittle-Peto alpha spending function[21]The DSMB will have unblinded access to all data and will discuss the results of the interim analysis with the steering committee in a joint meeting. The DSMB will advise the steering committee on continuation of the trial and the potential inclusion of additional participants based on the promising zone sample size re-

DocuSign Envelope ID: E42CD872-B126-44E1-9888-9E11235E4D76

estimation, after which the steering committee will decide on the continuation of the trial. If stopped prematurely, this decision will be reported to the central ethics committee.

8.5.1 Specific issues

The health issues under study are several postoperative complications after cardiac surgery that are related to incomplete wound drainage and retained blood. Excessive blood loss and/or cardiac tamponade are the most important complications and surgical reinterventions because of these are therefore primary endpoint. There are three issues that need the attention of the DSMB and the critical event committee (CEC):

1) The causes of postoperative bleeding are multifactorial and the pericardial flushing therapy will be effective for bleeding in case of a non-surgical cause. If there is a clear surgical cause (ie sutureline bleeding etc), CPPF therapy will have no effect on the bleeding. Although there is a definition for surgical bleeding in the study protocol, there may be cases in which it is not completely clear if the bleeding was surgical or not and this may be susceptible for interpretation and bias. This can have a direct effect on the primary endpoint outcome. The surgeon performing the redo surgery for bleeding is the first one to determine if the bleeding was surgical or not but like all SAE's, this will also be assessed by the clinical event committee (CEC). The clinical event committee will consist of an independent cardiothoracic surgeon and an intensivist or anaesthetist.

2) In case of a cardiac tamponade it does not matter if the bleeding cause is surgical or not; CPPF therapy can still prevent the onset of the tamponade because it enhances the evacuation of blood and clots from the pericardial space. But in this case the definition and diagnosis of cardiac tamponade may be susceptible for interpretation and bias. Also these events need to be assessed by the CEC.

3) Although very clear in the majority of cases, the decision to do or the timing of a surgical reintervention for bleeding is also dependent on other factors then bleeding rate alone, like clotting status and hemodynamic factors. This may be susceptible for interpretation and bias; the CEC should assess if there was a clear indication for surgical intervention or not. On the other hand, if there was excessive bleeding in the study group this should be noted and the CEC should assess if it was a rightly decision not to intervene. Following from this, clear definitions of surgical or non-surgical bleeding, excessive bleeding and cardiac tamponade are needed. A list of definitions will be provided in the protocol.

Following from our experience in the previous studies, a specific issue of CPPF therapy that needs to be addressed is "negative blood loss"; it was observed that in some cases flushing fluid was left behind in the patient, most probably absorbed in body cavities. While blood loss in the study group was calculated by extracting the inflow volume from the outflow volume (drainage production), this sometimes led to no, or even negative, blood loss, which is of course non-existent after cardiac surgery. This is also the main

DocuSign Envelope ID: E42CD872-B126-44E1-9888-9E11235E4D76

reason that CPPF therapy can only be used safely in daily clinical practice if we analyse the outflow volume for blood content by means of continuous haematocrit sensing and accurate in and outflow volume monitoring. This is precisely what the investigational device will be validated for in this study.

DocuSign Envelope ID: E42CD872-B126-44E1-9888-9E11235E4D76

9. STATISTICAL ANALYSIS

In this section we will describe the statistical analysis plan for the clinical report of the primary and secondary clinical end points of this study. The primary end point is the incidence of re-exploration for either cardiac tamponade and/or excessive bleeding due to non-surgical bleeding. The final study results will be presented following the CONSORT statement guidelines. For all hypothesis tests, two-tailed P values <0.05 will be considered statistically significant and no corrections will be made for multiple testing.

Handling of missing data

Missing data will be described per treatment group and will be reported in table footnotes to show the number of participants for whom the variable was missing. Data corrections will only be applied for parameters with <10% missing data. If outcome data could not be obtained at 3 months follow-up, we will first check the municipal council to ensure that the participant is not deceased. As a next step, the referring hospital will be contacted for follow-up information between randomization and six-month follow-up. All other participants are considered lost to follow-up. Although we expect the number of missing data to be low, if needed, multiple imputation techniques will be applied to account for missing data.

9.1 Primary study parameter(s)

The main analysis assesses superiority of the CPPF procedure compared to standard care for the primary end point: incidence of re-exploration for either cardiac tamponade and/or excessive bleeding due to non-surgical bleeding. Data will be presented per group as numbers with percentages and risk ratios with 95% Cis. Differences in the incidence of the primary outcome, i.e., the number of re-exploration for cardiac tamponade and/or excessive bleeding due to non-surgical bleeding, between both allocation arms will be assessed using a log-binomial regression model resulting in a risk ratio and accompanying 95% confidence interval (CI).

9.2 Secondary study parameter(s)

Secondary outcome data will be presented as numbers and percentages for categorical variables and as mean with standard deviation or median and interquartile range for continuous variables, as appropriate. Dichotomous secondary outcomes will be assessed using a log-binomial regression model resulting in a risk ratio and accompanying 95% confidence interval (CI). Differences in continuous outcomes between both allocation arms will be analysed using linear regression (normally distributed data) or quantile regression (non-normally distributed data) resulting in a mean or median difference, respectively, with accompanying 95% CI.

Blood loss

Actual blood loss is calculated by subtracting the total infused CPPF volume from the total MCTD volume after 8-hour stay in the ICU; no corrections will be made for patients with negative actual blood loss. This end point analysis will be supported by a sensitivity analysis that explores the influence of opened pleural cavities on (negative) actual blood loss.

DocuSign Envelope ID: E42CD872-B126-44E1-9888-9E11235E4D76

Missing data (<10% missing data) related to actual blood loss (at all other time points except after T8 hours) will be imputed using the last observation carried forward (LOCF) principle. Since blood loss is usually minimal, or ceased, 8 hours after surgery, hourly blood loss is not always accurately recorded in daily practice. When blood loss is not registered at T8, the last recorded blood loss is usually comparable to the blood loss at T8.
Delta haemoglobin is calculated between the last available intraoperative haemoglobin value and the haemoglobin level at 8-hour stay in the ICU. Transfusion requirements (red cells, fresh-frozen plasma, platelet concentrate) are defined as the number of units required per patient and between groups after randomization using a Poisson regression analysis.

Validation of the Haermonics investigational device sensors
The comparability of the DATAmed Obba hct sensors and Demcon hct sensor with the locally analyzed samples, as the comparability of the intrapericardial pressure sensor of the Haermonics investigational device with the central venous pressure sensor will be assessed using Bland-Altman plots.

Costs
Data will be presented as numbers and percentages for categorical variables and as mean with standard deviation or median and interquartile range. Incomplete data will be imputed with multiple imputation techniques. QALYs will be corrected for baseline utility if a difference in baseline utility value exist. Then, we will estimate the incremental cost per additional QALY gained for the Haermonics compared to standard care. Results will be presented using incremental cost-effectiveness planes and cost-effectiveness acceptability curves. Bootstrapping will be performed in order to assess the uncertainty around the cost difference between the two groups.

9.3 Other study parameters

Adverse events
All adverse events will be presented per group as numbers with percentages and risk ratios with 95% Cis.

Baseline characteristics
Baseline clinical characteristics and operative data will be presented per treatment allocation, and definitions will be presented in footnote items. Baseline characteristics will be described as numbers and percentages for categorical variables and as mean with standard deviation or median and interquartile range for continuous variables, as appropriate. Missing baseline data will be imputed using multiple imputation. Analysis of baseline data in relation to the primary outcome will be performed using a univariate linear regression model. Association of correlation will be explained for highly correlated parameters only and will be presented with a p-value

Assess potential impact of sample size re-estimation

DocuSign Envelope ID: E42CD872-B126-44E1-9888-9E11235E4D76

The main analysis will be performed separately in the participants included before and after the interim analysis and potential heterogeneity as a result of the sample size re-estimation will be assessed in the treatment effect across both periods.

9.4 Interim analysis

A promising zone sample-size re-estimation will be performed after 75% of participants have completed their 1-week follow-up period to assess the primary outcome. In addition, a formal interim analysis is planned to assess efficacy and safety of the treatment. A detailed description is provided in section 8.5.

DocuSign Envelope ID: E42CD872-B126-44E1-9888-9E11235E4D76

10. ETHICAL CONSIDERATIONS

10.1 Regulation statement

The study will be conducted according to the principles of the Declaration of Helsinki (2013, October 2013) and in accordance with the Medical Research Involving Human Subjects Act (WMO) and local hospital requirements.

10.2 Recruitment and consent

Patients meeting the inclusion criteria are invited to participate in the trial during their visit to the preoperative outpatient clinic or, if a patient is hospitalized and not able to visit the outpatient clinic, patients will be invited to participate one day prior to surgery. Patients will receive oral and written information about the study, and they will be given an informed consent form by a research assistant. One day preoperatively the patient will have the opportunity to ask questions about the study. At the end of the day, they will be asked to sign informed consent.

10.3 Benefits and risks assessment, group relatedness

The development of expected adverse events, fluid retention and infection (in pleural and pericardial cavities), will be closely monitored via several imaging techniques and laboratory measurements. The blood samples necessary for the study are not expected to negatively influence the result of treatment. A possible benefit for a patient receiving CPPF might be reduced postoperative blood loss as observed in previous trials.

10.4 Compensation for injury

The sponsor/investigator has a liability insurance which is in accordance with article 7 of the WMO.

The sponsor (also) has an insurance which is in accordance with the legal requirements in the Netherlands (Article 7 WMO). This insurance provides cover for damage to research subjects through injury or death caused by the study.
The insurance applies to the damage that becomes apparent during the study or within 4 years after the end of the study.

10.5 Incentives

No specific incentives, compensation or treatment will be received by the patients through participation in the study.

11. ADMINISTRATIVE ASPECTS, MONITORING AND PUBLICATION

11.1 Handling and storage of data and documents

All data are coded by a patient identification number (PIN). The password protected electronic subject identification log is stored separate from other study related datasets on the site network folder. Recorded data will be stored securely for 15 years in the archives. Data will be accessible only by the principal, coordinating and executive investigators. The requirements of the General Data Protection Regulation (GDPR, in dutch: Algemene Verordening Gegevensbescherming will be met.

11.2 Monitoring and Quality Assurance

The conduct of the study will be monitored by monitors from the Clinical Monitoring Center (CMC), location AMC. After approval of the study by the METC the executive researcher and/or the principal investigator will be invited for an intake interview. A study-specific monitor plan is drawn up by the CMC based on the intake. The monitoring plan specifies which activities will be carried out during the study period and when the monitor visits are planned. The number of visits is in accordance with the NFU guideline and depends, among other things, on the study risk, the vulnerability of the patient population and the duration of the study.

11.3 Amendments

A ‘substantial amendment’ is defined as an amendment to the terms of the METC application, or to the protocol or any other supporting documentation, that is likely to affect to a significant degree:

- the safety or physical or mental integrity of the subjects of the trial;
- the scientific value of the trial;
- the conduct or management of the trial; or
- the quality or safety of any intervention used in the trial.

All substantial amendments will be notified to the METC and to the competent authority.

Non-substantial amendments will not be notified to the accredited METC and the competent authority, but will be recorded and filed by the sponsor.

11.4 Annual progress report

The sponsor/investigator will submit a summary of the progress of the trial to the accredited METC once a year. Information will be provided on the date of inclusion of the first subject, numbers of subjects included and numbers of subjects that have completed the trial, serious adverse events/ serious adverse reactions, other problems, and amendments.

11.5 Temporary halt and (prematurely) end of study report

The sponsor will notify the accredited METC and the competent authority of the end of the study within a period of 90 days. The end of the study is defined as the last patient’s last visit.

DocuSign Envelope ID: E42CD872-B126-44E1-9888-9E11235E4D76

The sponsor will notify the METC immediately of a temporary halt of the study, including the reason of such an action.

In case the study is ended prematurely, the sponsor will notify the accredited METC and the competent authority within 15 days, including the reasons for the premature termination.

Within one year after the end of the study, the investigator/sponsor will submit a final study report with the results of the study, including any publications/abstracts of the study, to the accredited METC and the Competent Authority.

11.6 Public disclosure and publication policy

The Investigators are entitled to disseminate the findings of the study via publications in reputable scientific journals and via presentations at seminars or scientific conferences. The Investigators carry final responsibility for the scientific content of the publication on the main findings of the study.

DocuSign Envelope ID: E42CD872-B126-44E1-9888-9E11235E4D76

12. STRUCTURED RISK ANALYSIS

12.1 Potential issues of concern

In the process to obtain technical approval for use if the Haermonics investigational device, a structured risk analysis was performed, according to Medical Device Regulation (MDR) guidelines. In appendix D2, the investigational medical device dossier (IMDD) are details of the risk analysis and mitigation actions.

12.2 Synthesis

The Risk Analysis conclusions worksheet in the Risk Management File contains the following risk items and their mitigations:

Description of the risk	Comments on potential acceptance / non-acceptance
Electrical hazard (1,01 – 1,05)	In order to secure basic safety of the Haermonics investigational device, the device is designed according to the requirements of the IEC 60601-1 ed.3.1. Hazards described in this section refers to the possibility of coming in contact with live parts. Since device complies to requirements of 60601-1, all accessible parts are safe to touch (both for operator and patient) and live parts (above 24V) are not accessible without a tool.<br>Risk of the electrical hazard due to the short circuit caused by the spillage is mitigated by the design, as the electrical circuit and fluid circuit are physically separated from each other to minimalize possibility of the liquid ingress on the EL parts of the device. Haermonics investigational device incorporates several CE certified devices (UPS, medical grade Infuse pump, heater and drain), which will be used within their specification and which are integrated within the system in a manner which does not negatively impact their safety or conformance.<br>After the system integration, electrical safety according to IEC 60601-1 will be tested at DEMCON along with the functional tests in order to verify the effective implementation of the mitigation risks.<br>Residual risk of electrical hazard due to the operator mistake (e.g. 1,03) is minimalized by the fact that highly trained operators are responsible for the operation. Maintenance and service on the device is done by qualified personnel of Haermonics. Due to the taken mitigation actions and taking the risk-benefit analysis into account, remaining risk is considered to be acceptable (under the condition that the electrical safety test results are positive, and approved by the responsible person in the clinical institution where the investigational device will be used).
Electrical hazard due to use of the BF applied part (1,09)	The applied part of the infusion heater is classified as body floating (BF) and therefore it is possible that patient comes in contact with higher leakage currents (than in CF type part). In order to mitigate this risk, Leakage current will be measured in order to verify that it is lower than 10 microamperes. Additionally, probability of such risk is estimated as very low, as infusion heater disposable does not make direct contact to the heart but is connected with the heart via fluid column of saline solution, which is similar to a connection via the blood circulation system (the intended use of the infusion heater). Furthermore, previous clinical tests have also been performed

	using a BF infusion heater and in none of those cases, this hazardous situation occurred. Therefore, remaining risk is considered to be acceptable.
Instability hazard (2,01)	Risk of the device falling due to the instability is mitigated by the design of the device, so that it complies to the requirements of the clause 9.4 of the IEC 60601-1. Additionally, during the transport of the device during the treatment, device will be fastened to the bed so that the risk of the device falling is negligible. After the transport, device will be placed next to bed and the wheels of the trolley shall be blocked with the brakes in order to avoid accidental movement.<br>After taking into the consideration taken mitigation actions and risk benefit analysis, remaining risk is considered to be acceptable.
Suffocation hazard (2,04)	As the device contains tubing that is required for the intended use of the device, risk of the suffocation is present. However, used tubing are short as possible and the instruction for use contains warning about the risk. Additionally, placement of the tubing and the device with respect to the patient will also minimalize the possibility of the entanglement (tubing will not be placed above the neck). As the patient after the surgery will be under the supervision of the medical personnel aware of this risk, it is considered to be highly unlikely that the tubing of the device could get entangled around the patient's neck leading to suffocation. Furthermore, patients after the cardiac surgery will be sedated and connected to the respiratory equipment. Therefore, remaining risk is considered to be acceptable.
Mechanical hazard leading to tubing coming loose (2,05; 2,06)	In the case that the tubing would be pulled with high force (e.g. by the patient or other person pulling on the tubing or caused by the accidental pushing the device away from the bed), inflow and/or outflow tubing could get ripped from the patient's body. In the worst-case scenario, as a result patient will require an immediate operation.<br>In order to minimalize this risk, during the transport of the device during the treatment, device will be fastened to the bed so that the risk of pushing the device away is negligible. After the transport, device will be placed next to bed and the wheels of the trolley shall be blocked with the brakes in order to avoid accidental movement. Furthermore, tubing from patient to device will be secured to the y splitter (e.g. with a tie wrap).<br>As the risk of tubing coming loose is well known and accepted risk related to tubing and it is not a specific risk introduced by the Haermonics investigational device, remaining risk is considered to be acceptable.
Infection hazard (3,13; 4,07)	Risk of the exposure to the contaminated drain fluid as a result of the incorrect handling of the drain pods (e.g. forgetting to place them or to replace them on time) is also mitigated by the extended training of the device operator, who is aware of the risk. The operator responsible for the preparation for the treatment and the exchange of the drain pods, will supervise the state of the patient and in case of the leakage of the drain fluid, will act according to the hospital safety protocol, therefore the remaining risk is considered to be acceptable.
Usability / user error hazard (4,03; 5,23)	In case of the operator mistake, wrong flushing solution (other than saline) can be used for the purpose of treatment, which may result in the heart failure (depending on the type of the substance). However as only an adequately trained operator is

DocuSign Envelope ID: E42CD872-B126-44E1-9888-9E11235E4D76

	allowed to prepare the system for the treatment and as all bags of the saline solution are placed at once at the beginning of the treatment, therefore no exchange of bags during the treatment is required), it is highly unlikely that the operator, aware of critical importance of the treatment procedure would use other substances for the purpose of the infuse and the remaining risk is considered to be acceptable.<br><br>Risk of forgetting to connect the drain compartment may lead to the incorrect estimation of the blood loss (medical personnel estimates the blood loss based on the amount of the drain fluid). This risk is mitigated by the extended training of the device operator, that is aware of the risk. Operator responsible for the preparation for the treatment and the exchange of the drain canisters, will supervise the state of the patient and in case of the leakage of the drain fluid, will act according to the hospital safety protocol. Use of drains is common in the hospital ICU setting, therefore no additional risk is introduced. Medical personnel is aware that the drain canisters needs to be observed and replaced. Therefore the remaining risk is considered to be acceptable.
Mechanical hazard due to accidental movement of tubing (4, 13; 4,14)	Risk of the accidental movement is mitigated by use tubing as short as possible and securing the tubing from patient to the device to the y splitter. Furthermore, during the transport device will be fastened to bed and otherwise, brakes on the wheels shall be locked to avoid accidental movement of the device (and therefore tubing).<br>Additionally, this risk is not specific to the Haermonics investigational device and the risk is a well-known and accepted consequence of inflow/drainage tubing as a part of the cardiac surgery procedures.<br>Also, risk of the accidental removal of other patient leads (e.g. pacing leads) is well known and accepted risk while removing drain tubing after the surgery and is not an extra risk introduced by the Haermonics investigational device.<br>Therefore the remaining risk is considered to be acceptable.
Insufficient drainage leading to cardiac tamponade or damage to the heart or pericardium (4,06; 5,02; 5,04; 5,10)	Insufficient drainage of the fluid can be caused by erratic working of the pump or blockage of the drain system.<br>In case that the drain fluid cannot be removed from the patients pericardium, pressure in the pericardium could build up leading to the hazardous situation for the patient. In order to minimalize this risk, Haermonics investigational device contains a passive overflow circuit. In case that the pressure in the system rises, excess of the drain fluid will leave the system into the overflow pods before the pressure in the pericard will rise into a hazardous level. In order to mitigate this risk, the existing pressure measurement (Central Venous Pressure) will be used for monitoring of the pressure, so the hazardous situation can be detected. Due to the nature of the mitigation actions, remaining risk is considered to be acceptable.<br>If the outflow line would disconnect in such a way that the drain fluid would not be able to be flushed from the patient pericard, pressure in the pericard could rise to a dangerous levels. In order to mitigate this risk, the existing pressure measurement (Central Venous Pressure) will be used for monitoring of the pressure, so the hazardous situation can be detected. Additionally, state of the patient after the

DocuSign Envelope ID: E42CD872-B126-44E1-9888-9E11235E4D76

	surgery will be periodically checked by an operator aware of the this risks. Therefore, remaining risk is considered to be acceptable.
Foreseeable misuse (5,09)	As the values measured by the device will be displayed in the GUI of the investigational device, medical personnel might be willing to use the values in order to make clinical decisions. However, as one of the goals of this clinical trial is to verify effectiveness of the measurements performed by the device, medical personnel will be fully aware that the shown values shall not be used for the purpose of making clinical decisions. Additionally, GUI will contain a warning that the displayed values shall not be used for the clinical purposes. Also, trained personnel will as a standard not take clinical decisions (re-operations due to high pressure or excessive blood loss) based on one input parameter only. As only highly trained personnel will be responsible for the device and interaction with the patient, remaining hazard is considered to be acceptable.
Disruption of intended use (5,13a; 5,13b 5,14; 5,25; 5,26; 5,27; 5,28)	In case that the inflow pump would deliver to high or to low flow or too high pressure, that would impact the performance of the device leading to the incorrect pressure at the patient pericard and ineffective therapy.<br>In order to mitigate this risk, the existing pressure measurement (Central Venous Pressure) will be used for monitoring of the pressure, so the hazardous situation can be detected. Additionally, if the pressure within the system rises, excess of the drain fluid will leave the system into the overflow pods before the pressure in the pericard will rise into a hazardous level. Additionally, only highly trained operators will be responsible for the preparation of the device for the therapy, including setting the correct values of the infuse pump. State of the patient will be monitored and the operator can act in case of the therapy parameters are not optimal.<br>Therefore, after considering the implemented mitigation actions and the risk-benefit analysis, remaining risk is considered to be acceptable.
Incorrect vacuum level (5,19; 5,20; 5,21)	An incorrect vacuum level in the system might lead to the buildup of the pressure in the pericardium (due to too low suction) or to too much suction (due to the too high vacuum level), which might lead to the damage of the heart or pericardium. In order to mitigate this risk, only CE approved vacuum regulator (not part of the Haermonics device) shall be used during the therapy, Additionally, the state of the patient will be monitored on regular bases, so that the medical personnel can act in case emergency.<br>As the vacuum regulators are used as a standard in drainage devices and maintained by the ICU, it is expected that the chance of failure leading to a hazardous situation is negligible and the remaining risk is considered to be acceptable.
Thermal hazard (6,01)	Inflow fluid temperature being either too high or too low can lead to the disturbance of the heart rhythm of the patient. However, as the used infuse system allows setting the temperature only within the range limited to 39 ±2 C , which does not lead to a hazardous situation for the patient. Additionally, the state of the patient will be monitored and therefore, operator can act in case of the device malfunction.<br>Therefore, after considering the implemented mitigation actions and the risk-benefit analysis, remaining risk is considered to be acceptable

DocuSign Envelope ID: E42CD872-B126-44E1-9888-9E11235E4D76

EMC hazard (7,02)	In order to minimalize the risk that the Haermonics investigantional device would be source of an EMC disturbance capable of disturbing the working of surrounding devices, the Haermonics investigational device will be tested against the requirements of the IEC 60601-1-2 ed. 4.0 for emission. (Note per 17-Feb2021: The results of this test and the corresponding report will be submitted for approval to the responsible person at the AMC, and only after this approval is received the system is allowed for use in clinical practice.)<br>Therefore, at this stage (17-Feb-2021) the implemented mitigation actions need to be verified (EMC test) before the risk can be considered acceptable.

Overall conclusion of the risk analysis

All of the recognized risks have been either mitigated to the Low level (acceptable according to the risk criteria in Risk Management Plan) or to Medium level, in which case, acceptability has to be analyzed. All remaining Medium risks have been analyzed and are considered to be acceptable, taking into account the benefit risk analysis of the device and the nature of the both risk and risk control activities and actions (mostly based on design according to the harmonized standards e.g. EN 60601-1, 60601-1-2).

According to the criteria of the overall risk acceptability, taking into account the analysis of the medium risks in the section above and the overall residual risks resulting from the combined medium and low risks described in this document, it is concluded that the overall residual risk is acceptable as the benefits of this investigational study outweigh the remaining risks.

The final implementation of the risk mitigation actions and the conclusion on the acceptance of the remaining risks through benefit risk analysis, shall be reviewed after the verification of the device.

13. REFERENCES

1. Diephuis E, de Borgie C, Tomšič A, Winkelman J, van Boven WJ, Bouma B, et al. Continuous postoperative pericardial flushing method versus standard care for wound drainage after adult cardiac surgery: A randomized controlled trial. EBioMedicine. 2020;55:102744.
2. Diephuis EC, de Borgie CA, Zwinderman A, Winkelman JA, van Boven WP, Henriques JPS, et al. Continuous postoperative pericardial flushing reduces postoperative bleeding after coronary artery bypass grafting: A randomized trial. EClinicalMedicine. 2021;31:100661.
3. Brown JA, Kilic A, Aranda-Michel E, Navid F, Serna-Gallegos D, Bianco V, et al. Long-Term Outcomes of Reoperation for Bleeding After Cardiac Surgery. Semin Thorac Cardiovasc Surg. 2020.
4. Fitzgerald J, McMonnies R, Sharkey A, Gross PL, Karkouti K. Thrombin generation and bleeding in cardiac surgery: a clinical narrative review. Can J Anaesth. 2020;67(6):746-53.
5. Tirilomis T, Bougioukas IG, Friedrich MG, Danner BC, Schoendube FA. Re-exploration Early after Cardiac Surgery in Adults: The Importance of Bleeding-Related Complications. Heart Surg Forum. 2020;23(2):E174-e7.

DocuSign Envelope ID: E42CD872-B126-44E1-9888-9E11235E4D76

6. Gozdek M, Pawliszak W, Hagner W, Zalewski P, Kowalewski J, Paparella D, et al. Systematic review and meta-analysis of randomized controlled trials assessing safety and efficacy of posterior pericardial drainage in patients undergoing heart surgery. The Journal of thoracic and cardiovascular surgery. 2017;153(4):865-75.e12.
7. Karimov JH, Gillinov AM, Schenck L, Cook M, Kosty Sweeney D, Boyle EM, et al. Incidence of chest tube clogging after cardiac surgery: a single-centre prospective observational study. European journal of cardio-thoracic surgery : official journal of the European Association for Cardio-thoracic Surgery. 2013;44(6):1029-36.
8. Butts B, Goeddel LA, George DJ, Steele C, Davies JE, Wei CC, et al. Increased Inflammation in Pericardial Fluid Persists 48 Hours After Cardiac Surgery. Circulation. 2017;136(23):2284-6.
9. Guan X, Li J, Gong M, Lan F, Zhang H. The hemostatic disturbance in patients with acute aortic dissection: A prospective observational study. Medicine (Baltimore). 2016;95(36):e4710.
10. Kremke M, Tang M, Bak M, Kristensen KL, Hindsholm K, Andreasen JJ, et al. Antiplatelet therapy at the time of coronary artery bypass grafting: a multicentre cohort study. European journal of cardio-thoracic surgery : official journal of the European Association for Cardio-thoracic Surgery. 2013;44(2):e133-40.
11. Poon SS, Estrera A, Oo A, Field M. Is moderate hypothermic circulatory arrest with selective antegrade cerebral perfusion superior to deep hypothermic circulatory arrest in elective aortic arch surgery? Interactive cardiovascular and thoracic surgery. 2016;23(3):462-8.
12. Weber C, Gassa A, Rokohl A, Sabashnikov A, Deppe AC, Eghbalzadeh K, et al. Severity of Presentation, Not Sex, Increases Risk of Surgery for Infective Endocarditis. The Annals of thoracic surgery. 2019;107(4):1111-7.
13. Yamabe T, Zhao Y, Sanchez J, Kelebeyev S, Bethancourt CR, McMullen HL, et al. Probability of Uneventful Recovery After Elective Aortic Root Replacement for Aortic Aneurysm. The Annals of thoracic surgery. 2020;110(5):1485-93.
14. Manshanden JS, Gielen CL, de Borgie CA, Klautz RJ, de Mol BA, Koolbergen DR. Continuous Postoperative Pericardial Flushing: A Pilot Study on Safety, Feasibility, and Effect on Blood Loss. EBioMedicine. 2015;2(9):1217-23.
15. Kara H, Erden T. Feasibility and acceptability of continuous postoperative pericardial flushing for blood loss reduction in patients undergoing coronary artery bypass grafting. Gen Thorac Cardiovasc Surg. 2020;68(3):219-26.
16. Ali JM, Gerrard C, Clayton J, Moorjani N. Reduced re-exploration and blood product transfusion after the introduction of the Papworth haemostasis checklist†. Eur J Cardiothorac Surg. 2019 Apr 1;55(4):729-736. doi: 10.1093/ejcts/ezy362. PMID: 30346507.
17. Agarwal S, Choi SW, Fletcher SN, Klein AA, Gill R; Contributors. The incidence and effect of resternotomy following cardiac surgery on morbidity and mortality: a 1-year national audit on behalf of the Association of Cardiothoracic Anaesthesia and Critical Care. Anaesthesia. 2021 Jan;76(1):19-26. doi: 10.1111/anae.15070. Epub 2020 May 13. PMID: 32406071.

DocuSign Envelope ID: E42CD872-B126-44E1-9888-9E11235E4D76

18. Knapik P, Cieśla D, Saucha W, Knapik M, Zembala MO, Przybyłowski P, Kapelak B, Kuśmierczyk M, Jasiński M, Tobota Z, Maruszewski BJ, Zembala M; KROK Investigators. Outcome Prediction After Coronary Surgery and Redo Surgery for Bleeding (From the KROK Registry). J Cardiothorac Vasc Anesth. 2019 Nov;33(11):2930-2937. doi: 10.1053/j.jvca. 2019.04.028. Epub 2019 May 2. PMID: 31281012.
19. St-Onge, S., Chauvette, V., Hamad, R. et al. Active clearance vs conventional management of chest tubes after cardiac surgery: a randomized controlled study. J Cardiothorac Surg 16, 44 (2021).
20. NHR data: https://www.hartenvaatcijfers.nl/storage/reports/2022/nhr-rapportage-2022.pdf
21. Mehta CR, Pocock SJ. Adaptive increase in sample size when interim results are promising: a practical guide with examples. Stat Med. 2011 Dec 10;30(28):3267-84. doi: 10.1002/sim.4102. Epub 2010 Nov 30. PMID: 22105690.
22. Collette, L. Sample Size Re-Estimation as an Adaptive Design. Applied Clinical Trials-09-01-2021, Volume 30, Issue 9

DocuSign Envelope ID: E42CD872-B126-44E1-9888-9E11235E4D76

14. APPENDIX

1. Table 1 - SPIRIT diagram
2. Figure 1 - technical overview Haermonics investigational device

L4. List of prespecified SAEs

D2. IMDD

Table 1

SPIRIT figure indicating visits/study-specific assessments.

Study period	Screening	OR	ICU	ICU/Ward	Follow-up
Visit	outpatient clinic or 1 day prior to surgery				
Time, day	pre-op	0	ICU admission	post-op day 3	3 month post-op
In-/exclusion criteria	X				
Informed consent	X				
Demographics/history	X				
Quality of life (EQ-5D)	X			X	X
OR data (incl. CPB, cross-clamp, operating times)		X			
**STANDARD DIAGNOSTICS:**					
Lab markers	X		X	X	
X-ray	X		X	X	
ECG	X		X	X	
TTE*	X			X	X
**STUDY SPECIFIC BLOOD SAMPLING**:					
Systemic		X	X		
Pericardial		X			
Chest tube			X		
Outcomes: ICU mortality, hospital mortality, 30-day mortality, length of ICU stay, length of hospital Stay, adverse events		X	X	X	X
Concomitant medication	X	X	X	X	

* For some CABG patients, 3 day postoperative TTE is not according to standard protocol
