# Supplementary material for: Effects of Continuous Postoperative Pericardial FLUshing with Investigational Device on Postoperative Re-Explorations for Bleeding (FLUID)—Randomized Clinical Trial
Source: J Clin Med. 2026 Mar 11;15(6):2151. doi: 10.3390/jcm15062151 (PMC13026474; doi:10.3390/jcm15062151)
Supplement: Supplementary file 1 [file jcm-15-02151-s001.zip › jcm-4113872-supplementary/Statistical Analysis Plan - FLUID study - 04012026.pdf]

Statistical Analysis Plan

# Continuous Postoperative Pericardial Flushing After General Cardiac Surgery Procedures, With the Haermonics Investigational Device (FLUsh with Investigational Device, FLUID)—a randomized clinical trial


Manon A. Molenaar[1,2], Dave R. Koolbergen[2,3], Arend de Weger[3], Kayan Lam[4], Erik N. Hofman[5], Robert J.M. Klautz[1,2], Marcus J. Schultz[1]

**Amsterdam University Medical Centers, location AMC, Amsterdam, The Netherlands:**
[1]Department of Intensive Care
[2]Department of Cardiothoracic Surgery

**Leiden University Medical Center, Leiden, The Netherlands:**
[3]Department of Cardiothoracic Surgery

**Catharina Hospital, Eindhoven, The Netherlands:**
[4]Department of Cardiothoracic Surgery

**St. Antonius Hospital, Nieuwegein, The Netherlands:**
[5]Department of Cardiothoracic Surgery

**Correspondence:**
Manon A Molenaar
Department of Intensive Care
Amsterdam UMC, location AMC
Amsterdam, The Netherlands
Email: m.a.molenaar@amsterdamumc.nl

## INTRODUCTION

The randomized clinical trial named ‘FLUsh with Investigational Device’ (FLUID) compared continuous postoperative pericardial flushing (CPPF) with standard postoperative care in patients after cardiothoracic surgery with use of cardiopulmonary bypass.

The primary objective of this study was to determine the effect of CPPF therapy, executed by the Haermonics investigational device (Haermonics, Eindhoven, The Netherlands) on incidence of surgical re–exploration for either cardiac tamponade or excessive bleeding due to a non–surgical cause within the first postoperative week. Secondary objectives were to investigate the effect of CPPF on postoperative blood loss, transfusion of blood products and coagulation factors, postoperative atrial fibrillation, and hospital length of stay. In addition, the study investigated safety and feasibility of the Haermonics investigational device, and validated its hematocrit– and pressure sensors, explored the effect of CPPF on intraluminal chest tube clogging, and the cost–effectiveness of CPPF.

The study was planned to enroll a total of 992 patients (496 in each arm). Enrollment of patients was prematurely stopped because the study did no longer meet the original objectives. Preliminary findings of the study have led to several improvements of the medical device and its usage, and it was deemed necessary to design a new study that will use the improved version of the device.

This is an updated and final statistical analysis plan of FLUID, drafted and finalized before cleaning and closing of the study database. This analysis focuses on the clinical objectives, i.e., the effect of CPPF on the incidence of surgical re–exploration, its effects on postoperative blood loss and postoperative transfusion of blood products and coagulation factors, next to safety and feasibility of the Haermonics

investigational device. Validation of the hematocrit– and pressure sensors, and the effects of CPPF on intraluminal chest tube clogging will be analyzed separately. A statistical analysis plan for cost–effectiveness of CPPF will also be reported elsewhere.

## METHODS

### *Design*

FLUID is an investigator –initiated national, multicenter, randomized clinical superiority trial performed in the Amsterdam University Medical Centers, Amsterdam, The Netherlands, the Leiden University Medical Center, Leiden, the Catharina hospital, Eindhoven, and the St. Antonius hospital, Nieuwegein. The study protocol of FLUID was approved by the Institutional Review Board of the Amsterdam University Medical centers, location Academic Medical Center (2021_057). The study is registered at clinicaltrials.gov (study identifier NCT05308589). Written informed consent was obtained in all patients before surgery.

At the moment FLUID stopped, a total of 164 patients were randomized and treated.

### *Patients*

Patients were eligible for participation if: (1) aged > 18 years; (2) scheduled for a general cardiothoracic surgical procedure with use of cardiopulmonary bypass; and (3) having provided written informed consent. We excluded patients with a Euroscore II > 20%; patients undergoing emergency surgery; patients undergoing off–pump surgery or minimal invasive surgery; and patients participating in another study. Patients were additionally excluded in case of intraoperative diaphragm injury.

### *Randomization and blinding*

Randomization was done using random block sizes of 4 to 8 patients and stratifying per center with a password protected, web–based randomization system (SSL–encrypted website, Castor Electronic Data Capture, Amsterdam, the Netherlands). Because of the nature of the intervention, blinding was not possible.

*Intervention*

Patients randomized to the standard care group received one chest tube in the pericardial space and one chest tube in the anterior mediastinum after surgery, according to local standard protocols. Additionally, each surgically opened pleural cavity was drained separately. All chest tubes were connected to each other with the use of a Y-piece.

Patients randomized to the study group received, in addition to the standard of care, CPPF therapy during the first 8 postoperative hours. At sternal closure an extra infusion tube was inserted through an extra incision hole and positioned in the pericardial space. This extra infusion tube was directly connected to a bag of irrigation solution (NaCl 0,9%) and runs through a volumetric pump and through a heating device. CPPF was started at sternal closure and was performed continuously at a fixed flow rate of 500ml/hour, until the irrigation volume of 4000ml had been infused.

*Outcomes*

The primary outcome is the incidence of surgical re–exploration for either cardiac tamponade or excessive postoperative bleeding due to a non–surgical cause within the first postoperative week. Surgical re–exploration for another reason does not count herein. The re-explorations that occurred in the study were objectively assessed by the blinded clinical event committee. The reason for re-exploration was determined based on specific criteria: (1) presence of bleeding, (2) presence of hemodynamic instability,

(3) diagnosed cardiac tamponade, and (4) differentiation of surgical or non-surgical cause of the bleeding.

Secondary outcomes include blood loss within the first 8 postoperative hours, the transfusion of blood products and administration of coagulation factors in the first postoperative week. Other outcomes include new onset postoperative atrial fibrillation (POAF) within the first postoperative week, and length of stay in in the operating hospital. Initially, the intention was to also collect ICU length of stay, however this proved to be unattainable.

Safety outcomes include occurrence of the following adverse events: cardiac tamponade, re–exploration for surgical and non-surgical bleeding, minimal invasive interventions for fluid accumulation, infections, myocardial infarction, sternal dehiscence, delirium, acute renal insufficiency, postoperative atrial fibrillation and mortality.

*Cleaning and closing of the database*

Data collection and management were facilitated by using Castor Electronic Data Capture, a secure electronic data capture system (Castor). All data were entered, and discrepant or missing data regarding the endpoints mentioned above were resolved before a definite lock of the database.

*Missing data*

Missing data will be reported per treatment group and will be reported in table footnotes to show the number of participants for whom the variable was missing. Imputation is used for data with <10% missing data, but only for the secondary endpoint.

Unexpected changes in the study management have led to incomplete data collection for some of the predefined endpoints, including postoperative electro cardiography, ICU stay, hospital stay/adverse events occurring in the hospital after

discharge to the referring hospital and adverse events occurring during the three-month follow-up.

Clinical endpoints will be evaluated according to this statistical analysis plan. Endpoints regarding the safety and feasibility of the Haermonics investigational device, validation of the hematocrit– and pressure sensors will be evaluated and reported by the manufacturer.

*Calculations and data management*

Actual blood loss is calculated by subtracting the total infused CPPF volume from the total mediastinal chest tube drainage volume after 8–hour stay in the ICU; for patients with negative hourly blood loss corrections will be made as negative blood loss is not possible. In the calculation for the total blood loss, negative values of blood loss are not subtracted from the total, but instead, they are set to zero.

Missing data (<10% missing data) related to actual blood loss (at all other time points except after 8 hours) will be imputed using the last observation carried forward (LOCF) principle. Since blood loss is usually minimal, or ceased at 8 hours after surgery, hourly blood loss is not always accurately recorded in daily practice. When blood loss is not registered at 8 hours, the last recorded blood loss is usually comparable to the blood loss at 8 hours.

*Sample size*

The sample size for this study was based on an incidence of 4.5% for re–explorations for cardiac tamponade or excessive postoperative bleeding due to non–surgical bleeding[1–5], and the results of two earlier studies of CPPF suggesting a 70% reduction in the number of re–explorations[6–8]. A total of 992 study participants would have been needed. FLUID was prematurely stopped at 164 participants, and this number now serves as the sample size.

*Statistical analyses*

All statistical analyses will first be conducted on an intention–to–treat basis. Patients will be analyzed according to their assigned treatment arms.

Patient flow will be presented in a Consolidated Standards of Reporting Trials Diagram (CONSORT) diagram (**dummy Figure 1**).

Baseline characteristics and patient demographics (**dummy Table 1**), and perioperative data (**dummy Table 2**) will be presented as numbers and percentages for categorical variables, and as means with standard deviation or median and interquartile range for continuous variables, as appropriate.

Baseline characteristics and patient demographics will be compared using an unpaired t-test for continuous variables. For categorical variables a chi-squared test will be used to compare the groups.

The primary endpoint will be presented as the number per group of re–explorations for either cardiac tamponade or excessive bleeding due to a non–surgical cause and separately for cardiac tamponade or excessive bleeding due to a surgical cause. (**dummy Table 3**). Differences in the incidence of the primary outcome between both allocation arms will be assessed using a 2x2 contingency table resulting in a risk ratio and accompanying 95% confidence interval (CI). We will use a survival graph to display the probability of re–explorations occurring over time up to day 8 (**dummy Figure 2**).

Secondary outcome data will be presented as numbers and percentages for categorical variables and as mean with standard deviation or median and interquartile range for continuous variables, as appropriate (**dummy Table 3**). We will also use survival graphs to display the probability of secondary outcomes, where appropriate (**dummy Figure 3**). Categorical secondary outcomes will be assessed using a 2x2

contigency table resulting in a risk ratio and accompanying 95% confidence interval (CI). Differences in continuous outcomes between both allocation arms will be analyzed using unpaired t-test (normally distributed data) or Mann-Whitney U test (non-normally distributed data) resulting in a mean or median difference, respectively, with accompanying 95% CI.

All adverse events will be presented per group as numbers with percentages and risk ratios with 95% CIs (**dummy table 4**).

*Additional and subgroup analyses*

We will perform a per–protocol analysis by excluding patients who had one or more protocol violations. Patients assigned to the CPPF group will be excluded when they did not receive the therapy or if the therapy was halted before a minimum of six hours of flushing was completed. Patients assigned to the CPPF group will additionally be excluded if the therapy was halted for another reason than re-operation. Patients assigned to the control group will be excluded if they were not attached to the investigational device.

## REFERENCES


1. Knapik P, Cieśla D, Saucha W, et al. Outcome Prediction After Coronary Surgery and Redo Surgery for Bleeding (From the KROK Registry). *J Cardiothorac Vasc Anesth*. 2019;33(11):2930-2937. doi:10.1053/j.jvca.2019.04.028
2. Agarwal S, Choi SW, Fletcher SN, et al. The incidence and effect of resternotomy following cardiac surgery on morbidity and mortality: a 1-year national audit on behalf of the Association of Cardiothoracic Anaesthesia and Critical Care. *Anaesthesia*. 2021;76(1):19-26. doi:10.1111/anae.15070

3. Ali JM, Gerrard C, Clayton J, Moorjani N. Reduced re-exploration and blood product transfusion after the introduction of the Papworth haemostasis checklist†. *European Journal of Cardio-Thoracic Surgery*. 2019;55(4):729-736. doi:10.1093/ejcts/ezy362
4. Tirilomis T, Bougioukas IG, Friedrich MG, Danner BC, Schoendube FA. Re-exploration Early after Cardiac Surgery in Adults: The Importance of Bleeding-Related Complications. *Heart Surg Forum*. 2020;23(2):E174-E177. doi:10.1532/hsf.2893
5. Brown JA, Kilic A, Aranda-Michel E, et al. Long-Term Outcomes of Reoperation for Bleeding After Cardiac Surgery. *Semin Thorac Cardiovasc Surg*. 2021;33(3):764-773. doi:10.1053/j.semtcvs.2020.11.013
6. Manshanden JSJ, Gielen CLI, de Borgie CAJM, Klautz RJM, de Mol BAJM, Koolbergen DR. Continuous Postoperative Pericardial Flushing: A Pilot Study on Safety, Feasibility, and Effect on Blood Loss. *EBioMedicine*. 2015;2(9):1217-1223. doi:10.1016/j.ebiom.2015.07.031
7. Diephuis E, de Borgie C, Tomšič A, et al. Continuous postoperative pericardial flushing method versus standard care for wound drainage after adult cardiac surgery: A randomized controlled trial. *EBioMedicine*. 2020;55:102744. doi:10.1016/j.ebiom.2020.102744
8. Diephuis EC, de Borgie CA, Zwinderman A, et al. Continuous postoperative pericardial flushing reduces postoperative bleeding after coronary artery bypass grafting: A randomized trial. *EClinicalMedicine*. 2021;31:100661. doi:10.1016/j.eclinm.2020.100661

## DUMMY FIGURES

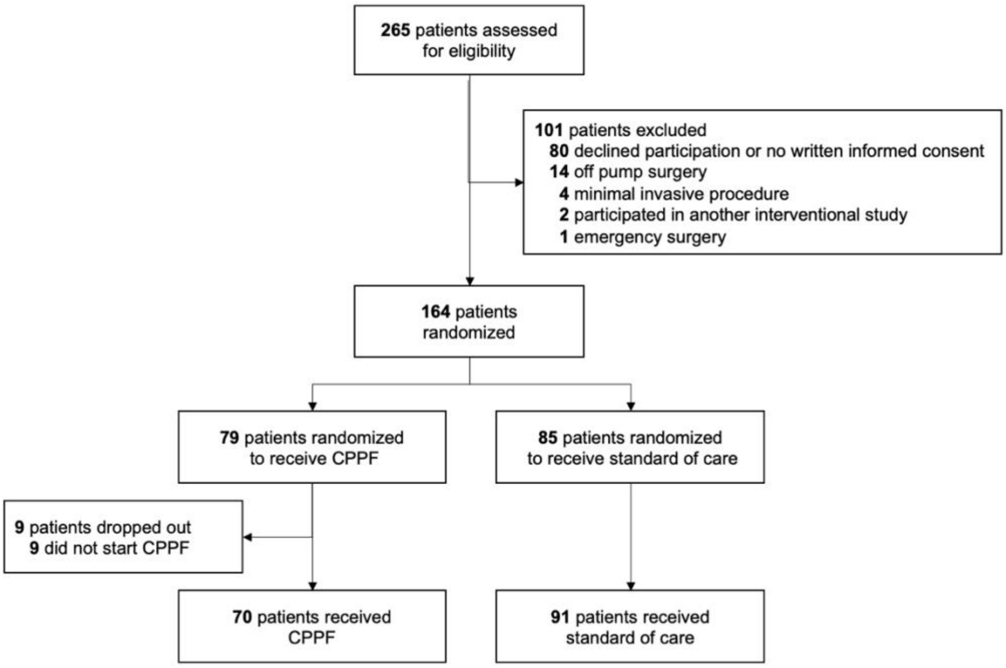


**Dummy figure 1: Patient flow chart (CONSORT-diagram)**

## DUMMY TABLES

**Dummy table 1 – Baseline characteristics of the patients**

| | CPPF (n = ..) | control (n = ..) | p-value |
|---|---|---|---|
| Age, years | | | |
| Sex, n (%) | | | |
| female | | | |
| male | | | |
| BMI, kg/$m^2$ | | | |
| Euroscore II | | | |
| Cardiovascular risk factors | | | |
| smoking (current/former) | | | |
| hypertension | | | |
| hypercholesterolemia | | | |
| diabetes mellitus | | | |
| myocardial infarction | | | |
| Co-morbidities | | | |
| atrial fibrillation | | | |
| micro- and macrovascular co-morbidity | | | |
| decompensation cordis | | | |
| pulmonary disorder | | | |
| neurological disorder | | | |
| renal disorder | | | |
| Malignancy | | | |
| Infection | | | |
| Surgical history | | | |
| cardiac surgery | | | |

| | | | |
|---|---|---|---|
| non-cardiac, thoracic surgery | | | |
| Left ventricular function | | | |
| Good | | | |
| Moderate | | | |
| Poor | | | |
| Preoperative anticoagulants | | | |
| none | | | |
| single | | | |
| double | | | |
| Type of preoperative anticoagulants | | | |
| platelet aggregation inhibitors | | | |
| heparins | | | |
| direct oral anticoagulants | | | |
| vitamin K antagonists | | | |
| Laboratory values | | | |
| hemoglobin (mmol/l) | | | |
| hematocrit (l/l) | | | |
| leukocyte count ($10^9$/l) | | | |
| platelet count ($10^9$/l) | | | |
| creatinine (μmol/l) | | | |
| INR | | | |
| CRP (mg/l) | | | |

Abbrevations: BMI, body mass index; INR, international normalized ratio; CRP, c-reactive protein.

**Dummy table 2 - Perioperative data**

| | CPPF n = .. (%) | control n = .. (%) | p-value |
|---|---|---|---|
| **number of surgical procedures** | …/… (…%) | …/… (…%) | |
| single procedure | …/… (…%) | …/… (…%) | |
| double procedure | …/… (…%) | …/… (…%) | |
| triple procedure | …/… (…%) | …/… (…%) | |
| procedures per patient median (IQR) | …/… (…%) | …/… (…%) | |
| **procedure type** | | | |
| coronary artery bypass grafting (CABG) | …/… (…%) | …/… (…%) | |
| valve surgery | …/… (…%) | …/… (…%) | |
| CABG combined with valve surgery | …/… (…%) | …/… (…%) | |
| aortic surgery | …/… (…%) | …/… (…%) | |
| reoperation | …/… (…%) | …/… (…%) | |
| **operative data** | | | |
| operation duration (min) | | | |
| number of surgically opened pleural cavities | …/… (…%) | …/… (…%) | |
| none | …/… (…%) | …/… (…%) | |
| one | …/… (…%) | …/… (…%) | |
| two | …/… (…%) | …/… (…%) | |
| cross-clamp duration (min) | …/… (…%) | …/… (…%) | |
| cardiopulmonary bypass duration (min) | …/… (…%) | …/… (…%) | |
| cell saver blood reinfused (ml) median (IQR) | …/… (…%) | …/… (…%) | |
| transfusion of blood products during surgery | | | |
| patients receiving PRBC, n/N (%) | …/… (…%) | …/… (…%) | |
| patients receiving plasma, n/N (%) | …/… (…%) | …/… (…%) | |
| patients receiving platelets, n/N (%) | …/… (…%) | …/… (…%) | |
| administration of coagulation factors during surgery | | | |
| patients receiving tranexamic acid n/N (%) | …/… (…%) | …/… (…%) | |
| patients receiving coagulation factors n/N (%) | …/… (…%) | …/… (…%) | |
| patients receiving fibrinogen n/N (%) | …/… (…%) | …/… (…%) | |

**Dummy table 3 – Primary and secondary outcomes**

| | CPPF (n = …) | control (n = …) |
|---|---|---|
| **primary endpoint** | | |
| re–thoracotomy | | |
| all, n/N (%) | …/… (…%) | …/… (…%) |
| for cardiac tamponade or excessive bleeding due to non–surgical bleeding, n/N (%) | …/… (…%) | …/… (…%) |
| for cardiac tamponade or excessive bleeding due to surgical bleeding, n/N (%) | …/… (…%) | …/… (…%) |
| for other reasons, n/N (%)[3] | …/… (…%) | …/… (…%) |
| **secondary endpoints** | | |
| blood loss, n/N[1] | ../.. | ../.. |
| total blood loss at 8-hours, ml, median [IQR] | .. (..-..) | .. (../..) |
| total blood loss until drain removal, ml, median [IQR] | .. (..-..) | .. (..-..) |
| blood loss > 0.5 L at 8–hours, n/N (%) | …/… (…%) | …/… (…%) |
| total blood loss > 1 L at 8–hours, n/N (%) | …/… (…%) | …/… (…%) |
| transfusion of blood products[2] | | |
| patients receiving PRBC, n/N (%) | …/… (…%) | …/… (…%) |
| patients receiving plasma, n/N (%) | …/… (…%) | …/… (…%) |
| patients receiving platelets, n/N (%) | …/… (…%) | …/… (…%) |
| units* in transfused patients | …/… (…%) | …/… (…%) |
| total number of PRBC units, median [IQR] | …/… (…%) | …/… (…%) |
| total number of plasma units, median [IQR] | …/… (…%) | …/… (…%) |
| total number of platelets units, median [IQR] | …/… (…%) | …/… (…%) |
| units* in all patients | …/… (…%) | …/… (…%) |
| total number of PRBC units, median [IQR] | …/… (…%) | …/… (…%) |
| total number of plasma units, median [IQR] | …/… (…%) | …/… (…%) |
| total number of platelets units, median [IQR] | …/… (…%) | …/… (…%) |
| new arrhythmias requiring medical intervention | …/… (…%) | …/… (…%) |
| postoperative atrial fibrillation, n/N (%) | …/… (…%) | …/… (…%) |
| other arrhythmias, n/N (%) | …/… (…%) | …/… (…%) |
| treatment for POAF | | |
| patients undergoing electrocardioversion | | |
| patients receiving antiarrythmic medication | | |
| wait and see policy | | |
| hospital stay length of stay (days) | | |
| **other endpoints** | | |
| thoracentesis, n/N (%) | …/… (…%) | …/… (…%) |
| pericardiocentesis, n/N (%) | …/… (…%) | …/… (…%) |
| pleuracentesis, n/N (%) | …/… (…%) | …/… (…%) |
| mortality | | |
| ICU mortality, n/N (%) | …/… (…%) | …/… (…%) |
| in–hospital mortality, n/N (%) | …/… (…%) | …/… (…%) |
| 30-days mortality | …/… (…%) | …/… (…%) |
| 180–days mortality, n/N (%) | …/… (…%) | …/… (…%) |

[1], follow–up not complete for all patients; [2], total number of transfusions after surgery during current hospital admission (i.e., not only in the acute phase after surgery); [3], for reasons like endocarditis (i.e., not in the acute phase)

*, units as provided by the local transfusion service

Abbreviations: n/N, numbers of patients; IQR, interquartile range; PRBC, packed red blood cells; POAF, postoperative atrial fibrillation; ICU, intensive care unit.

**Dummy table 4 - Adverse events**

| | hospital stay[1] | | | three-month follow-up[2] | | |
|---|---|---|---|---|---|---|
| | CPPF n = .. (%) | control n = .. (%) | p-value | CPPF n = .. (%) | control n = .. (%) | p-value |
| cardiac tamponade | …/… (…%) | …/… (…%) | | …/… (…%) | …/… (…%) | |
| acute tamponade | …/… (…%) | …/… (…%) | | …/… (…%) | …/… (…%) | |
| late tamponade | | | | | | |
| rethoracotomy | …/… (…%) | …/… (…%) | | …/… (…%) | …/… (…%) | |
| for non-surgical bleeding | …/… (…%) | …/… (…%) | | …/… (…%) | …/… (…%) | |
| for surgical bleeding | …/… (…%) | …/… (…%) | | …/… (…%) | …/… (…%) | |
| for other reasons | …/… (…%) | …/… (…%) | | …/… (…%) | …/… (…%) | |
| minimal invasive intervention for fluid accumulation | …/… (…%) | …/… (…%) | | …/… (…%) | …/… (…%) | |
| pericardial intervention | …/… (…%) | …/… (…%) | | …/… (…%) | …/… (…%) | |
| pleural intervention | …/… (…%) | …/… (…%) | | …/… (…%) | …/… (…%) | |
| infections | …/… (…%) | …/… (…%) | | …/… (…%) | …/… (…%) | |
| sepsis | …/… (…%) | …/… (…%) | | …/… (…%) | …/… (…%) | |
| pneumonia | …/… (…%) | …/… (…%) | | …/… (…%) | …/… (…%) | |
| pericarditis | …/… (…%) | …/… (…%) | | …/… (…%) | …/… (…%) | |
| deep sternal wound infection | …/… (…%) | …/… (…%) | | …/… (…%) | …/… (…%) | |
| surgical wound infection | …/… (…%) | …/… (…%) | | …/… (…%) | …/… (…%) | |
| sternal dehiscence | …/… (…%) | …/… (…%) | | …/… (…%) | …/… (…%) | |
| delirium | …/… (…%) | …/… (…%) | | …/… (…%) | …/… (…%) | |
| acute renal insufficiency | …/… (…%) | …/… (…%) | | …/… (…%) | …/… (…%) | |
| postoperative atrial fibrillation | …/… (…%) | …/… (…%) | | …/… (…%) | …/… (…%) | |
| myocardial infarction | …/… (…%) | …/… (…%) | | …/… (…%) | …/… (…%) | |
| mortality | …/… (…%) | …/… (…%) | | …/… (…%) | …/… (…%) | |

[1] data collection was limited to the operating center, [2] follow-up not complete for all patients

**Dummy figure 2: Survival graph primary outcome**

**Dummy figure 3: Survival graph secondary outcomes**
