# Supplementary material for: Effects of Continuous Postoperative Pericardial FLUshing with Investigational Device on Postoperative Re-Explorations for Bleeding (FLUID)—Randomized Clinical Trial
Source: J Clin Med. 2026 Mar 11;15(6):2151. doi: 10.3390/jcm15062151 (PMC13026474; doi:10.3390/jcm15062151)
Supplement: Supplementary file 1 [file jcm-15-02151-s001.zip › jcm-4113872-supplementary/Supplementary Tables S1 and S2 - proofread - MAMolenaar - 10032026.pdf]

Supplement to

Effects of Continuous Postoperative Pericardial FLUshing with Investigational Device on Postoperative Re-Explorations for Bleeding (FLUID)—Randomized Clinical Trial

Manon A. Molenaar[1,2], Dave R. Koolbergen[2,6], Martijn Vegter[2,6], Kayan Lam[4], Frederik N. Hofman[5], Stefan R. van Dinter[5], Annette van ‘t Loo[5], Arend de Weger[6], Jeroen A. Janson[7], Carolien S.E. Bulte[3], Susanne Eberl[3], Wim K. Lagrand[1], Alexander Vonk[2], Felice R.M. Lucas[1], Robert J.M. Klautz[2,6], Marcus J. Schultz[1]

Amsterdam University Medical Centers, Amsterdam, The Netherlands:
[1]Department of Intensive Care
[2]Department of Cardiothoracic Surgery
[3]Department of Anesthesiology

Catharina Hospital, Eindhoven, The Netherlands:
[4]Department of Cardiothoracic Surgery

St. Antonius Hospital, Nieuwegein, The Netherlands:
[5]Department of Cardiothoracic Surgery

Leiden University Medical Center, Leiden, The Netherlands:
[6]Department of Cardiothoracic Surgery
[7]Department of Intensive Care

Tables: 2

Correspondence:
Manon A Molenaar
Department of Intensive Care
Amsterdam UMC, location AMC
Amsterdam, The Netherlands

Email: m.a.molenaar@amsterdamumc.nl

Content

Table S1. Per-protocol analysis of primary endpoint. Page 3

Table S2. Post hoc analysis in high-risk patients. Page 4

Table S1. Per-protocol analysis of primary endpoint.

	CPPF (N = 74)	control (N = 85)	risk ratio (95% confidence interval)	*P*
**primary endpoint**				
re-thoracotomy				
for tamponade or excessive bleeding due to non-surgical cause	3/74 (4.1%)	2/85 (2.4%)	1.72 (0.30 – 10.03)	0.66
components of the primary endpoint*				
for tamponade	2/74 (2.7%)	1/85 (1.2%)	2.30 (0.21 – 24.83)	0.60
for excessive bleeding due to non-surgical cause	1/74 (1.4%)	2/85 (2.4%)	0.57 (0.05 – 6.21)	1.00

Data are numbers (%); *, re-thoracotomy could have been performed in patients for both reasons.
Abbreviations: CPPF, continuous postoperative pericardial flushing.

Table S2. Post hoc analysis in high-risk patients.

	CPPF (N = 29)	control (N = 24)	risk ratio (95% confidence interval)	*P*
**re-thoracotomy**				
for tamponade or excessive bleeding due to non-surgical cause	0/29 (0%)	2/24 (8.3%)	0.0 (0.0 - NaN)	0.20
components of this endpoint*				
for tamponade	0/29 (0%)	1/24 (4.2%)	0.0 (0.0 - NaN)	0.45
for excessive bleeding due to non-surgical cause	0/29 (0%)	2/24 (8.3%)	0.0 (0.0 - NaN)	0.20

Data are numbers (%); *, re-thoracotomy could have been performed in patients for both reasons.
Abbreviations: CPPF, continuous postoperative pericardial flushing.
