# Supplementary figures and images for: Effects of Continuous Postoperative Pericardial FLUshing with Investigational Device on Postoperative Re-Explorations for Bleeding (FLUID)—Randomized Clinical Trial
Source: J Clin Med. 2026 Mar 11;15(6):2151. doi: 10.3390/jcm15062151 (PMC13026474; doi:10.3390/jcm15062151)

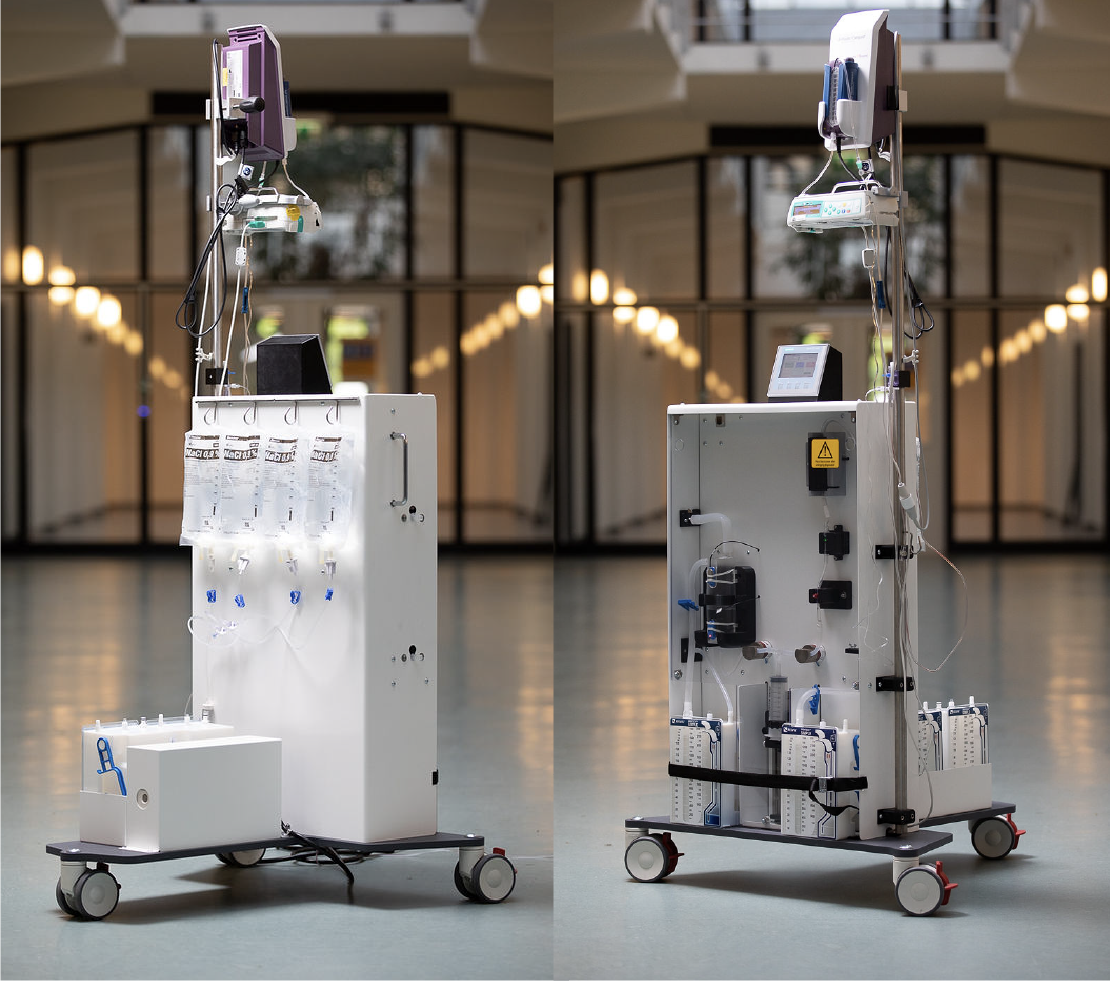

Supplement: Supplementary file 1 [file jcm-15-02151-s001.zip › jcm-4113872-supplementary/Figure S1 - Investigational Device.png]
